# Tianwang Buxin Dan regulated PI3K/Akt/mTOR signaling pathway to improve constipation and emotional disorders in rats

**DOI:** 10.3389/fmed.2026.1942569

**Published:** 2026-10-14

**Authors:** Heng Wang, Xinran Li, Zhaoyu Chen, Li Ji, Shaoliang Li, Haihua Qian, Dan Zhang

**Affiliations:** Department of Anorectal Surgery, The Affiliated Hospital of Nanjing University of Chinese Medicine, Nanjing, Jiangsu, China

**Keywords:** functional constipation, gut–brain axis, network pharmacology, PI3K/Akt/mTOR signaling, Tianwang Buxin Dan

## Abstract

**Background:**

Functional constipation (FC) is frequently comorbid with anxiety and depression, yet pharmacological strategies targeting both gastrointestinal and emotional symptoms remain limited. This study investigated the therapeutic effects and molecular mechanisms of Tianwang Buxin Dan (TWBXD), a classical Chinese herbal formula, in treating FC with emotional disturbances using an integrated strategy combining network pharmacology, molecular docking, and *in vivo* validation.

**Methods:**

TWBXD chemical constituents were characterized exclusively by UHPLC-MS/MS, yielding 650 compounds for network pharmacology. Targets were predicted via TargetNet and intersected with disease-associated genes from GeneCards. Common targets underwent PPI network analysis, GO/KEGG enrichment, and molecular docking. *In vivo*, a rat model was established by CUMS plus diphenoxylate and treated with TWBXD (low, medium, high doses) or positive drugs (mosapride plus fluoxetine) for 28 days. Therapeutic effects and pathway regulation in colon and hippocampus were evaluated by behavioral tests, gastrointestinal function assessment, histopathology, transmission electron microscopy, Western blotting, and qPCR.

**Results:**

Network pharmacology identified 473 drug targets and 308 disease-overlapping targets. KEGG enrichment highlighted PI3K-Akt, MAPK, IL-17, and neuroactive ligand–receptor interaction pathways, while PPI analysis identified Akt1, IL-6, and TNF as hub genes. Molecular docking showed quercetin, luteolin, and tanshinone IIA bound stably to Akt1, IL-6, and JUN (binding energies ≤ −6.6 kcal/mol). *In vivo*, TWBXD dose-dependently improved fecal water content, intestinal transit, sucrose preference, and open-field performance, and attenuated colonic mucosal injury, hippocampal neuronal damage, and mitochondrial disruption. Mechanistically, TWBXD was associated with increased PI3K/Akt/mTOR phosphorylation in colonic and hippocampal tissues and with reduced JNK expression in the hippocampus. No organ toxicity was observed.

**Conclusion:**

TWBXD alleviates constipation-related gastrointestinal dysfunction and comorbid anxiety/depression-like behaviors in rats, and these effects are associated with activation of PI3K/Akt/mTOR signaling in colonic and hippocampal tissues and with modulation of JNK signaling in the hippocampus. Although the concordance between computational predictions and multi-level *in vivo* validation supports TWBXD as a multi-target therapeutic candidate for functional constipation with emotional comorbidities, the causal contribution of PI3K/Akt/mTOR signaling, and the extent to which the peripheral and central changes are functionally coupled through the gut–brain axis, remain to be established.

## 1 Background

Functional constipation (FC) is a prevalent chronic gastrointestinal disorder characterized by infrequent defecation, hard stool consistency, and prolonged colonic transit time. Globally, its estimated prevalence is approximately 14%, with higher incidence observed among women and older adults (1). Beyond the physical discomfort, growing clinical and epidemiological evidence indicates that FC is frequently accompanied by emotional disturbances such as anxiety and depression. These psychological symptoms can, in turn, worsen intestinal dysfunction through the bidirectional gut–brain axis (2, 3). Pathophysiologically, the comorbidity involves disruptions in autonomic nervous regulation, neuroinflammation, microbiota imbalance, and impaired entero–central nervous system communication (4–6). However, conventional treatments such as laxatives and antidepressants often fail to provide lasting and comprehensive relief, highlighting the need for integrative, multi-target therapeutic strategies (7).

TWBXD is a classical Chinese medicinal formula composed of thirteen standardized herbal ingredients (Table 1). It was formulated under the principles of nourishing Yin, clearing internal heat, calming the mind, and moistening the intestines. TWBXD originates from *She Sheng Zong Yao* and has been further refined in modern clinical settings (8). Its major components include *Rehmannia glutinosa*, *Polygala tenuifolia*, *Ziziphus jujuba*, *Salvia miltiorrhiza*, and *Schisandra chinensis*, all of which have demonstrated neuroprotective, anti-inflammatory, anxiolytic, and gastrointestinal regulatory effects in pharmacological studies (9, 10). Clinically, TWBXD has been used to treat comorbid insomnia, anxiety, and constipation, but its underlying molecular mechanisms remain to be fully elucidated (11).

**Table 1:** The detailed information of TWBXD compositions.

Plant names	Chinese name	Medicinal part	Family	Weight Ratio
Rehmannia glutinosa (Gaertn.) Libosch. ex Fisch. & C.A.Mey	Sheng-Di-Huang	dried root	Orobanchaceae	20
Scrophularia ningpoensis Hemsl	Xuan-Shen	dried root	Scrophulariaceae	10
Salvia miltiorrhiza Bunge	Dan-shen	dried root	Lamiaceae	10
Platycodon grandiflorus (Jacq.) A.DC	Jie-Geng	dried root	Campanulaceae	10
Polygala tenuifolia Willd.	Yuan-Zhi	dried root	Polygalaceae	10
Prunus armeniaca L	Xing-Ren	seeds	Rosaceae	10
Angelica sinensis (Oliv.) Diels	Dang-Gui	dried root	Apiaceae	15
Schisandra chinensis (Turcz.) Baill	Wu-Wei-Zi	fruits	Schisandraceae	15
Ophiopogon japonicus (Thunb.) Ker Gawl	Mai-Dong	dried root	Asparagaceae	15
Asparagus cochinchinensis (Lour.) Merr	Tian-Dong	dried root	Asparagaceae	15
Platycladus orientalis (L.) Franco	Bai-Zi-Ren	seeds	Cupressaceae	15
Ziziphus jujuba Mill. var. spinosa (Bunge) Hu ex H.F.Chow	Suan-Zao-Ren	seeds	Rhamnaceae	15
Atractylodes macrocephala Koidz.	Bai-Zhu	dried root	Asteraceae	15

The name of all the plants in the article has been checked with https://www.worldfloraonline.org/.

The phosphoinositide 3-kinase/protein kinase B/mammalian target of rapamycin (PI3K/Akt/mTOR) signaling pathway plays a central role in modulating cell proliferation, synaptic plasticity, inflammatory response, and intestinal barrier function (12, 13). Dysregulation of this pathway contributes to both gut motility dysfunction and the development of mood disorders (14). In FC, suppressed PI3K/Akt/mTOR activity impairs interstitial cells of Cajal (ICCs), leading to colonic dysmotility and mucosal inflammation (15). Simultaneously, downregulation of this pathway has been linked to increased neuroinflammatory responses and reduced neurogenesis in mood disorders such as depression and anxiety (16, 17). As such, reactivation of the PI3K/Akt/mTOR axis offers a possible target for simultaneously addressing gastrointestinal and emotional dysfunction.

In light of the multifaceted nature of TWBXD and the complex pathophysiology of constipation with emotional disturbances, this study adopted an integrative strategy that combined network pharmacology, molecular docking, and *in vivo* experimental validation. This approach enabled the identification of active compounds and potential molecular targets of TWBXD and facilitated the mechanistic elucidation of its therapeutic effects via behavioral, histological, ultrastructural, and molecular biological analyses in a rat model. This study aims to provide a scientific foundation for further clarifying the pharmacodynamic substances and therapeutic mechanisms of TWBXD, ultimately supporting its clinical application in disorders involving the gut–brain axis, by integrating network pharmacology and molecular docking with multi-level *in vivo* validation in a comorbid constipation and emotional disturbance model and by providing parallel evidence from both colon and hippocampus.

## 2 Methods

### 2.1 Network pharmacology research

#### 2.1.1 Screening of active compounds and potential targets

The chemical constituents of TWBXD were characterized exclusively by UHPLC-MS/MS analysis of the TWBXD extract (Section 2.3). Compounds were identified using the MWDB (Metware Database) based on MS/MS spectral matching. To focus on exogenous phytochemicals with potential pharmacological relevance, primary endogenous metabolite categories, including amino acids and derivatives, nucleotides and derivatives, organic acids, lipids, saccharides, and vitamins, were excluded. Only compounds with identification Level 1 (MS/MS fragmentation and retention time match score ≥ 0.7) and Level 2 (match score 0.5–0.7) were retained, yielding a total of 1,513 exogenous phytochemicals. Compound names were standardized using PubChem (https://pubchem.ncbi.nlm.nih.gov/), and canonical SMILES strings were retrieved via the PubChem PUG REST API. Of these, 650 compounds with valid SMILES were used for subsequent analyses. Putative targets for each compound were predicted using the TargetNet web server (http://targetnet.scbdd.com/), which constructs multi-target QSAR models against 623 human proteins. Targets with a prediction probability score > 0.5 were retained and standardized to official gene symbols using the UniProt database (https://www.uniprot.org/), restricted to Homo sapiens (18). All computational analyses, including network pharmacology and molecular docking, were performed in accordance with the Standards for Computational Methods in Drug Design and Discovery recommended by the journal.

#### 2.1.2 Identification of disease related genes

Disease-related genes associated with functional constipation and emotional disorders were retrieved from GeneCards, DisGeNET, CTD and OMIM using the keywords “functional constipation,” “chronic constipation,” “anxiety,” “depression,” and “emotional disorder.” Genes with a relevance score above the median (>4.07) were retained, yielding 8,645 disease-associated genes after standardization and de-duplication. The final disease gene set was then used for intersection analysis with TWBXD-related targets (19, 20).

#### 2.1.3 Construction of compound–target–disease interaction network

The TWBXD related targets obtained in Section 2.1.1 and the disease related gene set obtained in Section 2.1.2 were intersected using the Venny 2.1 (https://bioinfogp.cnb.csic.es/tools/venny/index.html) online tool to identify common targets. These common targets were imported into Cytoscape version 3.10.2 (https://cytoscape.org/) to construct the compound–target–disease interaction network. Network topology features were analyzed using the Network Analyzer plugin to evaluate node importance and to identify key compounds and candidate targets within the pharmacological network (21).

#### 2.1.4 Protein–protein interaction (PPI) analysis

The common targets between TWBXD and the disease were uploaded to the STRING database (https://string-db.org/), setting the species as Homo sapiens and minimum required interaction score > 0.9. The resulting interaction network was downloaded in TSV format and visualized in Cytoscape. Hub targets were identified based on topological features such as degree and betweenness centrality (21). To improve the readability of the main figure, the complete PPI network is provided as Supplementary Figure S1, whereas Figure 2C displays a simplified core hub-target network highlighting the major high-connectivity targets.

#### 2.1.5 GO function and KEGG pathway enrichment analysis

Gene Ontology (GO) biological process annotation and Kyoto Encyclopedia of Genes and Genomes (KEGG) pathway enrichment were performed using the Metascape platform (https://metascape.org/). The *p*-value threshold was set at <0.01, and results were visualized in bar plots and bubble diagrams using R version 4.3.0 (22). Pathways for experimental validation were selected by integrating enrichment significance, PPI hub information, biological relevance to functional constipation and emotional disorders, and feasibility of validation in colonic and hippocampal tissues, rather than by enrichment rank alone.

#### 2.1.6 Molecular docking simulation

Core active compounds and key targets were selected for docking analysis. Targets for molecular docking were selected based on both PPI network topology and relevance to the signaling pathways evaluated *in vivo*. AKT1 and IL-6 were selected because they were among the top hub targets and represented the PI3K/Akt/mTOR-related and inflammatory/neuroimmune components, respectively. JUN was selected because it ranked among the top 10 PPI nodes in Supplementary Table S4 and is a canonical downstream effector of JNK/AP-1 signaling, which was evaluated experimentally in hippocampal tissue. INS, TNF, and TP53 were not prioritized for the present docking panel for the following reasons. INS encodes a secreted peptide hormone rather than a conventional intracellular small-molecule target. TNF was retained in the PPI and pathway analyses; however, to keep the docking panel focused and avoid redundancy among inflammatory cytokine targets, IL-6 was selected as a representative inflammatory/neuroimmune hub target for docking. TP53 is a pleiotropic nuclear transcription factor that was not directly evaluated in the *in vivo* validation experiments. Protein crystal structures were downloaded from the Protein Data Bank (https://www.rcsb.org/), and 2D molecular structures of compounds were retrieved from PubChem (https://pubchem.ncbi.nlm.nih.gov/). All ligands and receptors were prepared using AutoDock Tools version 1.5.7. Molecular docking simulations were performed using AutoDock Vina version 1.2. The strength of binding was evaluated by the binding energy, with a value less than −5.0 kcal/mol indicating strong binding affinity. Interaction models were visualized in PyMOL version 2.5.0 (23).

### 2.2 Animal experiments

#### 2.2.1 Ethics statement and animal housing conditions

All animal procedures complied with the “Guidelines for the Care and Use of Laboratory Animals” and were approved by the Animal Ethics Committee of the Affiliated Hospital of Nanjing University of Chinese Medicine (Approval No. 2023DW-051-02). A total of forty-eight specific-pathogen-free (SPF) Sprague–Dawley rats (half male and half female, 6 weeks old, weighing 200 ± 20 g) were provided by Jiangsu Huachuang Xinnuo Pharmaceutical Technology Co., Ltd. (license number SCXK (Su) 2020-0009). Rats were acclimatized for one week before modeling and housed under controlled conditions (20-25 °C, 30%–50% humidity) with a 12-h light/12-h dark cycle (lights on at 07:00 and off at 19:00), with food and water available *ad libitum*.

#### 2.2.2 Drug preparation and group assignment

TWBXD (batch number 220701) was prepared and provided by the Department of Pharmacy, the Affiliated Hospital of Nanjing University of Chinese Medicine, with the specific composition detailed in Supplementary Table 1. These herbs were soaked in six volumes of purified water for 30 min, boiled over high heat, then simmered for 40 min. The two decoctions were combined, filtered to remove residues, stored at 4 °C, and heated and mixed thoroughly before administration.

Mosapride citrate dispersible tablets (Fujian Haixi New Drug Creation Co., Ltd.; National drug approval number H20203264) were crushed and suspended in distilled water at a concentration of 0.135 mg/mL. Fluoxetine hydrochloride capsules (Shanxi QianYuan Pharmaceutical Group Co., Ltd.; National drug approval number H20073985) were crushed and suspended in distilled water at 0.158 mg/mL.

The carbon ink suspension used for gastrointestinal transit measurement was prepared by dissolving 100 g Arabic gum in 800 mL deionized water, adding 100 g powdered activated carbon, and boiling the mixture three times. After cooling, the solution was brought to a final volume of 1,000 mL with deionized water, stored at 4 °C, and thoroughly mixed before use.

According to *Pharmacological Experimental Methodology*, the equivalent conversion factor between humans and rats is 6.3 (24). The standard adult human clinical dose of TWBXD is 175 g/day for a 70 kg individual, corresponding to an equivalent rat dose of 15.75 g/kg/day. Based on this value, three intervention doses were selected: 0.5 × (7.88 g/kg/day), 1.0 × (15.75 g/kg/day), and 2.0 × (31.5 g/kg/day).

Six-week-old SPF rats (half male and half female) were acclimatized for one week prior to experimentation. Eight rats were randomly selected as the normal control group (Control), while the remaining 40 rats were subjected to a compound diphenoxylate administration combined with chronic unpredictable mild stress (CUMS) to establish a model of functional constipation with anxiety and depression. After successful modeling, the rats were randomly divided into five groups (*n* = 8): control group, model group, positive drug group (mosapride 1.35 mg/kg/d + fluoxetine group 1.58 mg/kg/d), TWBXD low-dose group (7.88 g/kg/day), TWBXD medium-dose group (15.75 g/kg/day), and TWBXD high-dose group (31.5 g/kg/day). All interventions were administered by intragastric gavage once daily for 28 consecutive days. A detailed description of the experimental grouping and drug administration is provided in Supplementary Table S1.

#### 2.2.3 Construction of functional constipation with emotional disturbance model

A compound rat model of functional constipation accompanied by emotional disturbances (e.g., anxiety and depression) was induced using a combination of chronic unpredictable mild stress (CUMS) and diphenoxylate intervention. The CUMS procedure lasted for six consecutive weeks and involved eleven classical stressors: 12-hour water deprivation, 12-hour food deprivation, 12-hour damp bedding exposure, swimming in 0 °C ice water for 3 min, 45° cage tilting, tail pinch for 1 min, 6-hour restraint, reversed light/dark cycle, overnight illumination, horizontal vibration for 5 min (160 Hz), and 6-hour noise exposure. Each day, one or two of the above stressors were randomly selected and applied in combination with solitary housing to prevent habituation and maintain unpredictability. The same stressor was not repeated on consecutive days (25, 26).

Beginning in the third week of modeling, rats were administered diphenoxylate orally at a dose of 10 mg/kg/day for 28 days to promote the onset of constipation symptoms. The total modeling duration was six weeks, during which animals were continuously exposed to both physical and pharmacological stressors.

Constipation was confirmed by measuring fecal water content and small intestinal propulsion rate, which were compared statistically with the control group. Emotional disturbance was evaluated based on typical behavioral and physiological symptoms, including coarse and dull fur, lethargy, reduced activity, decreased food and water intake, weight loss, reduced responsiveness, startle sensitivity, and diminished volitional behavior. Behavioral assessments such as sucrose preference and open field tests were used to validate the depression- and anxiety-like phenotypes. Successful model establishment was evaluated based on reductions in fecal output, fecal water content, and intestinal transit rate, together with behavioral abnormalities detected by OFT and SPT, as previously validated by our group (27).

#### 2.2.4 Behavioral assessments

Emotional behaviors were evaluated using the sucrose preference test (SPT) and open field test (OFT). In the SPT, animals were water-deprived for 22 h before exposure to 1% sucrose solution and distilled water for 1 h. Sucrose preference was calculated as the ratio of sucrose intake to total fluid intake. The OFT was conducted in a 100 × 100 × 40 cm square arena divided into central and peripheral zones. Animals were placed in the center, and their movement was recorded for 5 min. Total distance traveled, time spent in the central area, and frequency of center crossings were analyzed to assess exploratory and anxiety-like behaviors (28, 29).

#### 2.2.5 Assessment of gastrointestinal transit and defecation

Gastrointestinal function was assessed using three indices. The fecal water content was measured by weighing 24-hour fecal output before and after oven-drying at 110 °C for 3 h, and calculated as (wet weight – dry weight)/wet weight  × 100%.

To assess distal colonic transit, a 3-mm plastic bead was inserted 3 cm into the rectum, and the time to spontaneous expulsion was recorded.

For small intestinal motility, rats were assessed 30 min after gavage with 2 mL of charcoal suspension. Rats were anaesthetised using isoflurane inhalation (induction 4%–5%, maintenance 1.5%–2.5%). Rats were euthanized by exsanguination under deep isoflurane anesthesia. Death was confirmed by cessation of heartbeat and respiration. The small intestine was excised, and the distance traveled by the charcoal front relative to the full intestinal length was calculated as propulsion rate (%) = (charcoal distance/total length) × 100% (30).

#### 2.2.6 Histopathological and ultrastructural examination

Under isoflurane anesthesia (induction 4%–5%, maintenance 1.5%–2.5%), blood was collected from the abdominal aorta. Rats were euthanized by exsanguination under deep isoflurane anesthesia. Death was confirmed by cessation of heartbeat and respiration. A 10 cm segment of distal colon (starting 2 cm from the anus) was excised, rinsed with cold PBS (0.1 M, pH7.4, 4 ℃), and fixed in 4% paraformaldehyde. The brain was removed by enlarging the occipital foramen, and bilateral hippocampi were rapidly dissected (<3 min) based on the Paxinos and Watson atlas. Hippocampal tissues were snap-frozen in liquid nitrogen and stored at −80 °C.

Paraffin-embedded colon and hippocampal tissues were stained with hematoxylin–eosin (H&E) to assess inflammatory infiltration, mucosal and neuronal damage. Nissl staining was used to evaluate neuronal integrity in the hippocampal CA1 region under a light microscope.

Ultrastructural changes in mitochondria of colonic epithelial and hippocampal cells were observed using transmission electron microscopy (TEM) to assess treatment-related structural preservation across groups.

#### 2.2.7 Western blotting (WB)

The bicinchoninic acid (BCA) method (Beyotime, China) was used to quantify total protein extracted from colon and hippocampal tissues. In colonic tissue, PI3K p85, p-Akt, Akt, p-mTOR, mTOR, and GAPDH were examined; in hippocampal tissue, JNK, p-Akt, Akt, p-mTOR, mTOR, and GAPDH were examined. Proteins were separated by SDS-PAGE and transferred onto polyvinylidene difluoride membranes. After blocking with 5% nonfat milk, the membranes were incubated with primary antibodies overnight at 4 °C, followed by secondary antibodies. Protein bands were visualized using a Gel Imaging System (Alpha RED, USA). Western blot quantification was performed using three independent replicates per group. Representative blots are shown in Figure 10, and densitometric analyses were calculated from the three independent replicates. The relative expression levels of p-mTOR/mTOR and p-Akt/Akt were calculated, while PI3K p85 and JNK were normalized to GAPDH (31).

#### 2.2.8 Quantitative real-time PCR (qPCR)

Total RNA was extracted from colon and hippocampal tissues using TRIzol reagent (Vazyme, China). RNA concentration and purity were measured spectrophotometrically. Reverse transcription was performed using HiScript III RT SuperMix (Vazyme, China). Quantitative PCR was carried out using Taq Pro Universal SYBR Green Master Mix (Vazyme, China) on a CFX96 Real-Time PCR System (Bio-Rad, USA). Relative expression levels of PI3K, Akt, and mTOR mRNA in colonic tissue, and of Akt, mTOR, and JNK (Mapk8) mRNA in hippocampal tissue, were analyzed after normalization to GAPDH (32). Primer sequences for all genes, including JNK (Mapk8), are listed in Supplementary Table S2.

#### 2.2.9 Serum biochemical analysis

Drug-induced toxicity is typically dose-dependent and multi-organ targeted, often leading to pathological changes such as hepatocellular necrosis, renal tubular injury, and cardiomyocyte apoptosis. Serum biochemical markers serve as key indicators for evaluating systemic toxicity. In this study, blood samples were collected from rats in the high-dose TWBXD group (approaching the maximum tolerated dose) and the blank control group after 28 days of treatment. Liver function (ALT, AST), renal function (UREA, CREA), and cardiac enzymes (CK) were assessed using an automated biochemical analyzer. Normal reference ranges for each parameter are listed in Supplementary Table S3.

### 2.3 UHPLC-MS/MS analysis

The chemical constituents of TWBXD were analyzed using UHPLC-MS/MS. Electrospray ionization was applied in both positive and negative ion modes, and multiple reaction monitoring was used for compound annotation and relative quantification. Chromatographic separation was performed on an ExionLC AD system with an Agilent SB-C18 column (1.8 μm, 2.1 × 100 mm) at 40 °C, with an injection volume of 2 μL and a flow rate of 0.35 mL/min. The mobile phase consisted of water containing 0.1% formic acid (A) and acetonitrile containing 0.1% formic acid (B), using a gradient from 5% to 95% B (0–9.0 min), held at 95% B (9.0–10.0 min), returned to 5% B (10.0–11.1 min), and re-equilibrated to 14.0 min. Mass spectrometry was operated at 500 °C with ion spray voltages of 5,500 V (positive) and −4,500 V (negative). The widely targeted UHPLC-MS/MS analysis was used to characterize the chemical profile of TWBXD. The peak-area data from this analysis were used for compound annotation and semi-quantitative profiling, but were not treated as absolute contents. To provide quantitative information for representative marker constituents, eight selected compounds were further quantified by targeted multiple reaction monitoring (MRM) using authentic reference standards and external calibration curves. Calibration curves were constructed by linear regression of MRM peak areas against nominal concentrations with 1/x^2^ weighting. The selected marker constituents included ferulic acid, polygalaxanthone III, chlorogenic acid, rutin, naringenin, schisandrol A, luteolin, and hyperin. The MRM transitions, calibration ranges, correlation coefficients, and measured concentrations are provided in Supplementary Table S8.

### 2.4 Statistical analysis

All data were expressed as mean ± standard deviation (SD). For comparisons among multiple groups, one-way ANOVA was performed, followed by Tukey's multiple-comparison test for pairwise group comparisons (or Dunnett's test when comparing each treatment group with the model group). For outcomes measured at multiple time points, two-way repeated-measures ANOVA was applied, followed by Sidak–adjusted *post hoc* comparisons. Shapiro–Wilk and Levene's tests were used to assess normality and homogeneity of variance; when assumptions were violated, the Kruskal–Wallis test with Dunn's multiple-comparison test was used. Adjusted *P* < 0.05 was considered statistically significant. Statistical analyses were performed using IBM SPSS Statistics version 22.0. All graphical visualizations, including bar graphs, scatter plots, and line charts, were generated using GraphPad Prism 10.0.

## 3 Results

3

### 3.1 Chemical characterization of TWBXD

The chemical profile of TWBXD was characterized by UHPLC-MS/MS operated in multiple reaction monitoring mode under both positive- and negative-ion conditions. Representative chromatograms (Figure 1) showed abundant signals throughout the analytical run in both ion modes, indicating the chemical complexity of TWBXD. Compounds were annotated based on MS/MS spectral information and standardized to remove redundant entries and synonymous names. To focus on exogenous phytochemicals with potential pharmacological relevance, primary endogenous metabolite categories, including amino acids and derivatives, nucleotides and derivatives, organic acids, lipids, saccharides, and vitamins, were excluded, and only compounds with identification levels 1–2 were retained. After filtering, a total of 1,513 exogenous phytochemicals (Levels 1–2) were retained from UHPLC-MS/MS analysis. Of these, 650 compounds with valid PubChem SMILES structures were used as the input for subsequent network pharmacology and molecular docking analyses. The retained constituents covered major phytochemical classes, including flavonoids, phenolic acids, alkaloids, terpenoids, lignans/coumarins, and steroids. Representative constituents identified in TWBXD are summarized in Table 2. In addition to the representative constituents identified by widely targeted UHPLC-MS/MS, eight selected marker constituents were quantified by targeted MRM using external calibration curves. These marker constituents were selected according to the availability of authentic reference standards, their representativeness for the component herbs, and their coverage of major chemical classes in TWBXD, including phenolic acids, flavonoids, lignans, and characteristic constituents from Polygalae Radix and Schisandrae Chinensis Fructus. The quantitative results are shown in Supplementary Table S8.

**Figure 1:**
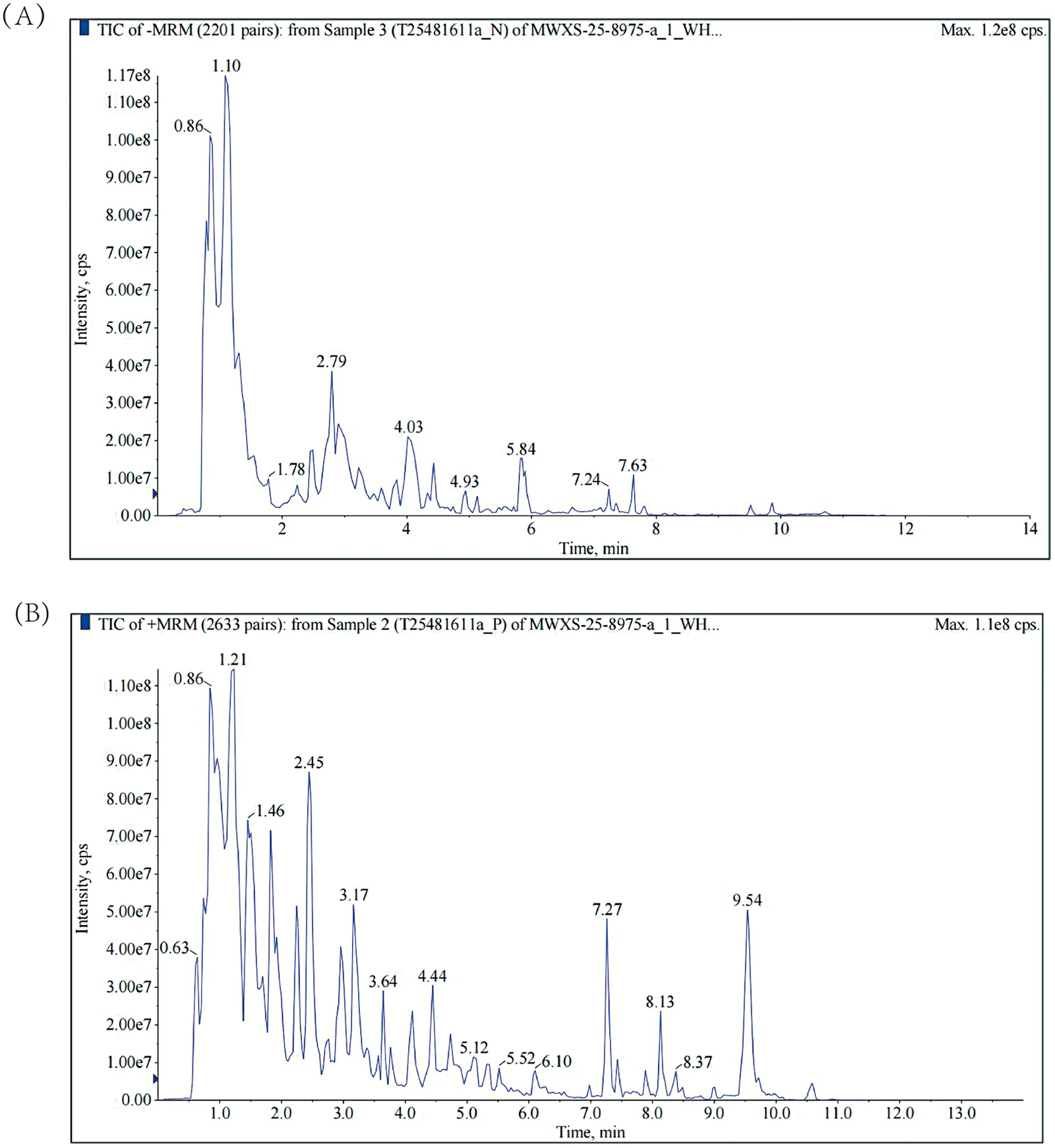
Representative TICs acquired in MRM mode for the TWBXD extract by UHPLC-MS/MS. **(A)** Negative ion mode. **(B)** Positive ion mode.

**Table 2:** Representative chemical constituents of TWBXD identified by UHPLC-MS/MS.

No.	Name	Formula	Class I	Q1	Q3	CAS	Level
1	Suberosin*	C15H16O3	Lignans and Coumarins	245.12	189.06	581-31-7	1
2	Corypalmine*	C20H23NO4	Alkaloids	342.17	297.11	13,063--54-2	1
3	Corydayanine	C20H20NO4+	Alkaloids	338.14	322.11	—	1
4	Methylene tanshinquinone	C18H14O3	Quinones	279.10	233.10	67,656--29-5	2
5	Hyperin*	C21H20O12	Flavonoids	463.09	301.04	482-36-0	1
6	Isoorientin	C21H20O11	Flavonoids	447.09	357.06	4261-42-1	2
7	13-Hydroxygermacrone*	C15H22O2	Terpenoids	235.17	179.11	—	1
8	6-methoxygeniposidic acid	C17H24O11	Others	405.14	151.04	—	1
9	10-Dehydrogeniposide	C17H22O10	Terpenoids	387.13	147.04	—	1
10	5,6,7-Trimethoxycoumarin*	C12H12O5	Lignans and Coumarins	237.07	176.04	55,085-47-7	1
11	Costunolide*	C15H20O2	Terpenoids	233.15	131.08	553-21-9	1
12	Alloimperatorin*	C16H14O4	Lignans and Coumarins	271.10	147.05	642-05-7	1
13	2′,4′-Dihydroxyacetophenone	C8H8O3	Others	153.05	65.04	89-84-9	1
14	Trillin-6′-O-glucoside*	C39H62O13	Steroids	739.42	577.37	—	1
15	Palmatrubine	C20H20NO4+	Alkaloids	338.14	322.10	16,176-68-4	1
16	Leucosceptoside A	C30H38O15	Phenolic acids	639.23	177.05	83,529-62-8	1
17	Pogostone	C12H16O4	Others	225.11	139.04	23,800-56-8	1
18	Columbamine*	C20H20NO4+	Alkaloids	338.14	323.11	3,621-36-1	1

Notes：Additional selected marker constituents quantified by targeted MRM are provided in Supplementary Table S8.

### 3.2 Potential therapeutic targets of TWBXD for functional constipation with emotional disorders: PPI network, enrichment analysis, and compound–target–disease network construction

To systematically elucidate the molecular mechanisms of TWBXD in the treatment of functional constipation with emotional disorders, a network pharmacology approach was employed. Active constituents were compiled from UHPLC-MS/MS experimental characterization exclusively. After name standardization and SMILES retrieval, a total of 650 compounds were subjected to TargetNet-based target prediction, yielding 473 unique TWBXD-related target genes after standardization to official gene symbols via UniProt.

Disease-related genes for functional constipation and emotional disorders were retrieved from GeneCards, DisGeNET, CTD, and OMIM and merged after gene symbol standardization and de-duplication, yielding 8,645 disease-related genes. Intersection analysis between TWBXD-related targets and disease-related genes identified 308 common targets, which were considered potential therapeutic targets of TWBXD and were used for downstream network construction and enrichment analyses (Figure 2A). These overlapping genes were used to construct a TWBXD compound–target interaction network. To improve visual clarity, the network was reorganized as an herb–compound–common target flow diagram, with herbs, compounds, and common targets arranged from left to right (Figure 2B). This layout preserved the source–compound–target relationships while reducing label overlap and network crowding. Several representative bioactive compounds, including luteolin, beta-sitosterol, quercetin, diosgenin, and rutin, were connected with multiple common targets, supporting their potential contribution to the pharmacological profile of TWBXD.

**Figure 2:**
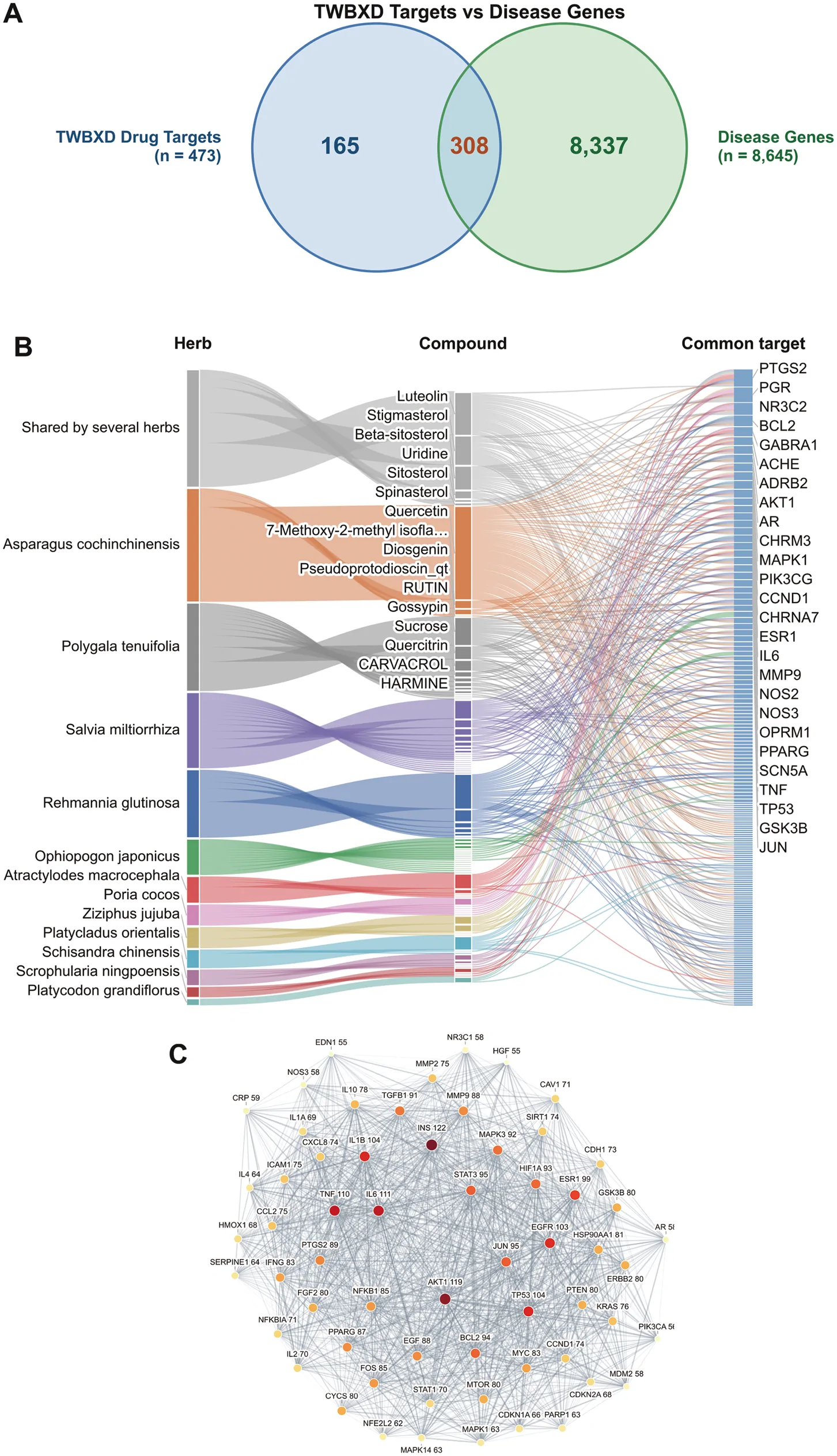
Network pharmacology analysis of TWBXD in functional constipation with emotional disorders. **(A)** Venn diagram showing 308 overlapping targets between 473 predicted TWBXD targets and 8,645 disease-related genes. **(B)** Herb–compound–common target flow diagram of TWBXD. Herbs, compounds, and common targets are arranged in the left, middle, and right columns, respectively. Each band represents a compound–target association, and band colour indicates the source herb. Representative high-connectivity compounds and common targets are labelled to improve readability. **(C)** Core hub-target network extracted from the PPI analysis. The labelled nodes represent high-connectivity targets, and the numbers after the gene symbols indicate their degree values. Node size and node colour reflect degree, and edges represent protein–protein associations among the displayed hub targets. The complete PPI network is provided in Supplementary Supplementary Figure S1.

To explore the interrelationships among the 308 common targets, a protein–protein interaction (PPI) network was constructed using the STRING database with a high-confidence interaction score (>0.9) and subsequently visualized in Cytoscape. The complete STRING PPI network is provided in Supplementary Figure S1. To improve readability in the main figure, the hub-target information was summarized as a simplified core network in Figure 2C, in which representative high-connectivity targets were labelled directly. Topological analysis identified several hub proteins, including AKT1, IL6, INS, TNF, TP53, STAT3, JUN, and EGFR, which may play pivotal roles in mediating the therapeutic effects of TWBXD through pathways involving inflammation, immune modulation, neuroendocrine regulation, and PI3K/Akt-related signaling. The degree and centrality information of the hub targets is summarized in Supplementary Table S4. Likewise, quercetin, luteolin, and beta-sitosterol emerged as representative active components of TWBXD (Supplementary Table S5), highlighting their potential contribution to the pharmacological effects against FC with emotional comorbidities.

### 3.3 Analysis of the biological processes and enrichment pathways associated with TWBXD treatment

To further elucidate the molecular mechanisms underlying TWBXD's therapeutic effects, Gene Ontology (GO) and Kyoto Encyclopedia of Genes and Genomes (KEGG) enrichment analyses were conducted on the 308 overlapping targets using the Metascape platform. The GO analysis revealed significant enrichment in biological processes (BP), cellular components (CC), and molecular functions (MF). The top BP terms included regulation of miRNA metabolic processes, transcriptional regulation by RNA polymerase II, and cytokine-mediated signaling. CC terms were mainly associated with chromatin structures, membrane rafts, and RNA polymerase II transcriptional regulatory complexes. MF terms were enriched in DNA-binding transcription factor activity and cytokine receptor interactions (Figure 3A).

**Figure 3:**
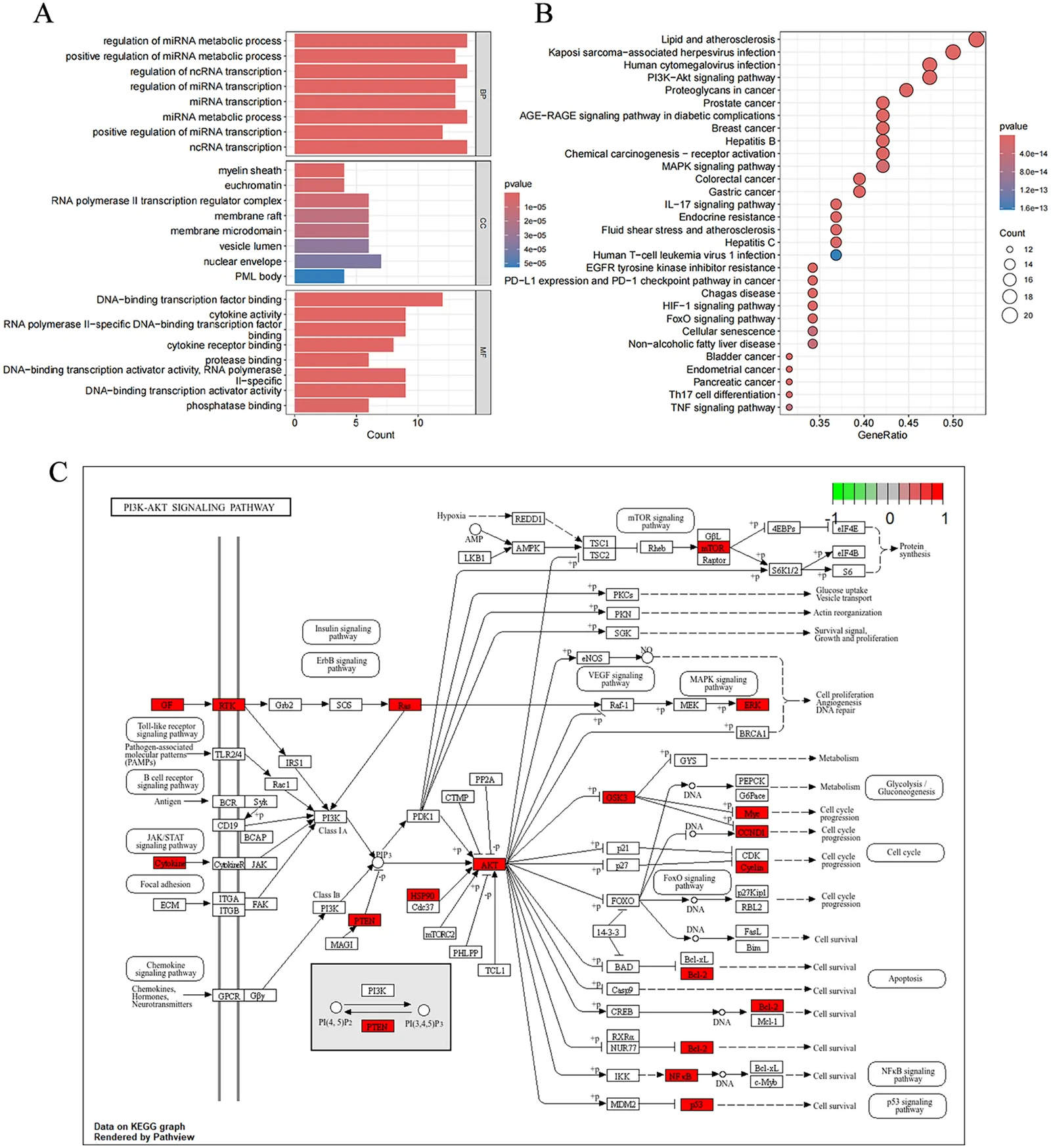
GO and KEGG enrichment analysis of common targets of TWBXD against FC with emotional disorders. **(A)** GO enrichment analysis of common targets, showing biological process (BP), cellular component (CC), and molecular function (MF) categories. **(B)** KEGG pathway enrichment analysis of common targets, showing significantly enriched pathways. **(C)** KEGG PI3K-Akt pathway map generated for the pathway selected for experimental validation, with TWBXD-related targets highlighted in red.

KEGG pathway analysis demonstrated that the common targets of TWBXD were involved in multiple signaling cascades, including MAPK signaling, IL-17 signaling, neuroactive ligand-receptor interaction, cancer-related pathways, and PI3K-Akt signaling (Figure 3B). Although PI3K-Akt signaling was not the highest-ranked KEGG term, it was significantly enriched and was selected for further experimental investigation based on biological relevance rather than enrichment rank alone. This pathway contains AKT1, one of the hub targets identified in the PPI analysis, and is closely connected with mTOR-mediated regulation of cell survival, inflammation, epithelial barrier integrity, intestinal motility, hippocampal neuroplasticity, and emotional regulation. Therefore, PI3K/Akt/mTOR signaling was considered a biologically plausible candidate pathway linking the network pharmacology results with the gastrointestinal and behavioral phenotypes observed in this study.

To better visualize TWBXD's modulation of the PI3K-Akt pathway, a pathway map was constructed by mapping the drug-affected targets onto the KEGG PI3K-Akt signaling diagram. The visualization highlighted the key regulatory nodes targeted by TWBXD (Figure 3C).

### 3.4 Molecular docking validation of key compounds and targets

To identify the most potent constituents of TWBXD, we docked all candidate compounds against three representative targets selected from the PPI hub and pathway-related targets, namely Akt1, IL-6, and JUN, using binding free energy ≤ −5.0 kcal mol⁻^1^ as the selection criterion. AKT1 and IL-6 were selected as representative hub targets, whereas JUN was included because of its close relationship with the JNK/AP-1 pathway assessed in the hippocampus. Docking calculations showed that quercetin, luteolin, and tanshinone IIA exhibited the strongest affinities, with scores ranging from −6.6 to −8.9 kcal mol⁻^1^. Tanshinone IIA produced the lowest (most favorable) energies for Akt1 (−8.6 kcal mol⁻^1^), IL-6 (−8.9 kcal mol⁻^1^), and JUN (−6.6 kcal mol⁻^1^), while quercetin and luteolin also surpassed the significance threshold (Table 3). The corresponding binding poses, illustrated in the newly labelled panels of Figures 4A to 4I, display hydrogen-bonding and hydrophobic interactions within the binding pockets of each target. These results suggest that quercetin, luteolin, and tanshinone IIA are likely the key pharmacodynamic constituents of TWBXD and may exert therapeutic effects by modulating Akt1-, IL-6-, and JUN-mediated neuroimmune-inflammatory pathways (Figure 4).

**Table 3:** Binding affinities of TWBXD active compounds with core targets.

Target	PDB ID	Quercetin (kcal/mol)	Luteolin (kcal/mol)	Tanshinone IIA (kcal/mol)
AKT1	8R5K	−6.7	−6.8	−8.6
IL-6	5GW9	−7.8	−8.3	−8.9
JUN	3S4E	−6.6	−6.6	−6.6

**Figure 4:**
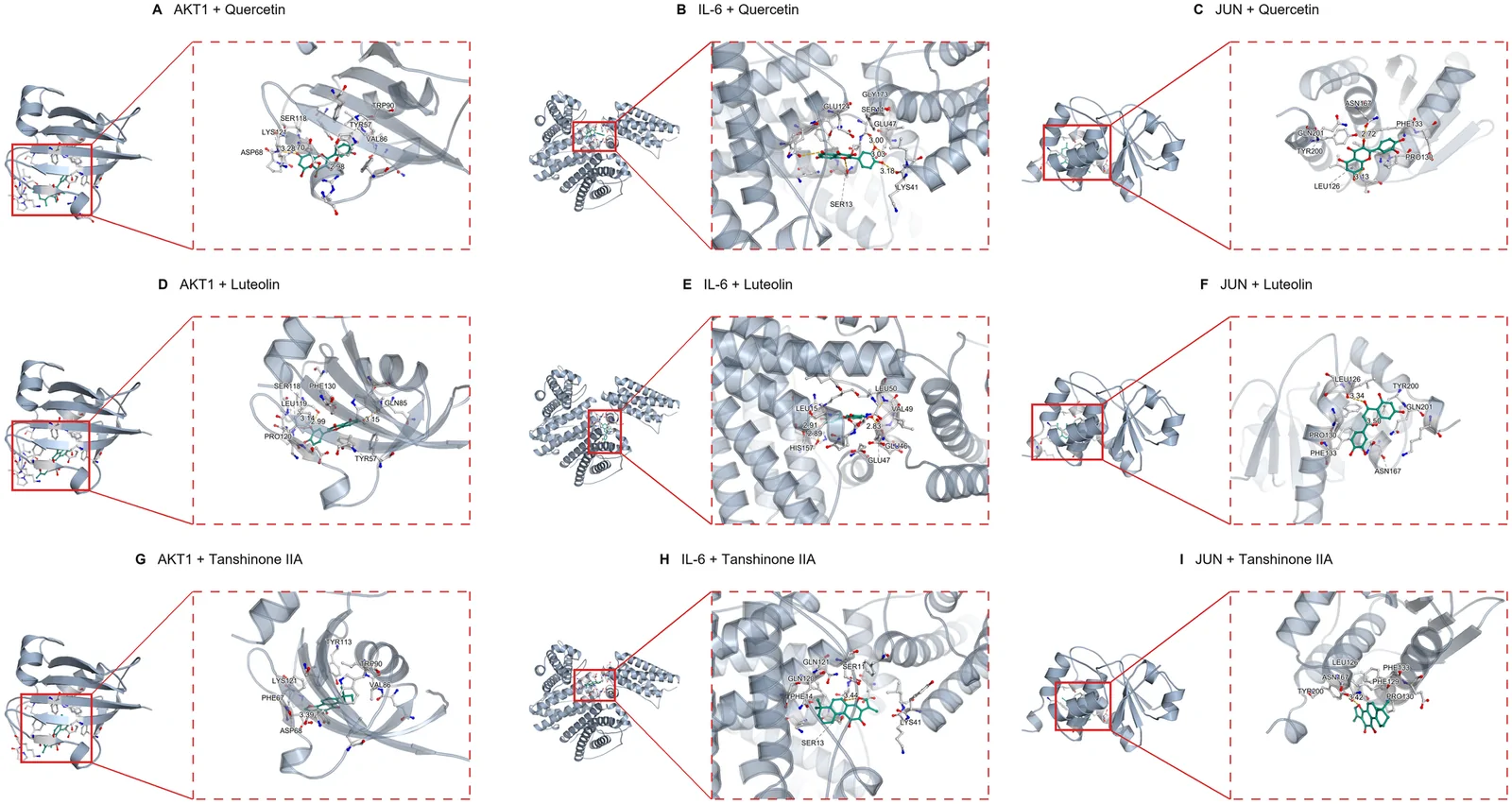
Molecular docking models between key compounds and selected core targets of TWBXD. Representative docking conformations are shown for the following protein-ligand pairs: **(A)** AKT1 + quercetin; **(B)** IL-6 + quercetin; **(C)** JUN + quercetin; **(D)** AKT1 + luteolin; **(E)** IL-6 + luteolin; **(F)** JUN + luteolin; **(G)** AKT1 + tanshinone IIA; **(H)** IL-6 + tanshinone IIA; **(I)** JUN + tanshinone IIA. The specific protein and ligand names are labelled directly above each panel. The left part of each panel shows the overall binding position, and the enlarged view shows the local binding pocket, interacting residues, and interaction distances. Ligands are shown as colored sticks, and protein structures are shown as grey cartoons.

### 3.5 TWBXD significantly increases fecal water content in rats with constipation and emotional disorders

Following model induction, rats exhibited signs of fatigue, poor grooming, hair loss, irritability, and reduced locomotion, indicative of successful establishment of the constipation model with comorbid emotional disorders. Compared with the control group, the model group showed a significant reduction in fecal water content (*p* < 0.01). After 14 and 28 days of treatment, TWBXD at medium and high doses significantly increased fecal water content compared to the model group (*p* < 0.05). The effect was dose-dependent, with the high-dose group showing the greatest improvement. These results suggest that TWBXD can effectively alleviate dry stools associated with constipation in a dose-dependent manner (Figure 5, Supplementary Table S6).

**Figure 5:**
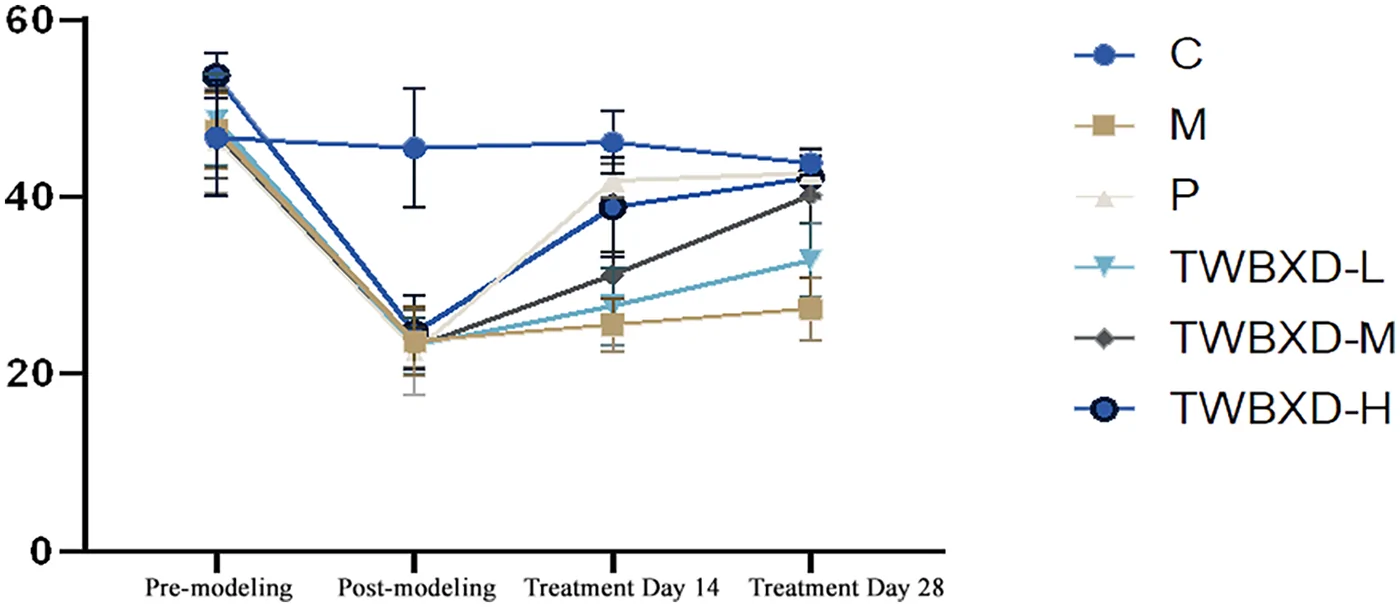
Changes in fecal water content in each group before and after treatment. C, control group; M, model group; P, positive drug; TWBXD-L/M/H, low, medium, and high doses of Tianwang Buxin Dan. Data are expressed as mean ± SD (*n* = 8). Statistical analysis was performed using two-way repeated-measures ANOVA followed by Sidak-adjusted *post hoc* comparisons. Significant pairwise comparisons are indicated in Supplementary Table S6. *P < 0.05 compared with the control group; #*P* < 0.05 compared with the model group.

### 3.6 TWBXD improves intestinal motility and carbon ink propulsion rate in constipated rats

In the model group, the time to first black stool and distal colonic transit time were significantly prolonged, while the carbon ink propulsion rate was markedly reduced compared with the control group (*p* < 0.01), indicating impaired intestinal transit. TWBXD treatment for 14 and 28 days significantly improved all three parameters across dose groups (*p* < 0.05, *p* < 0.01), showing a clear dose-dependent effect. These improvements reflect TWBXD's capacity to enhance gastrointestinal motility and colonic propulsion in rats with functional constipation (Figures 6A–6C, Supplementary Table S7).

**Figure 6:**
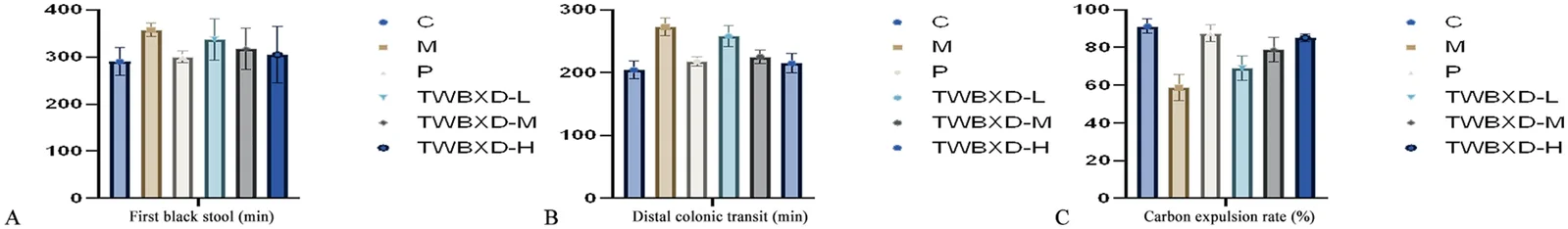
Effects of TWBXD on intestinal motility and carbon ink propulsion in rats. C, control group; M, model group; P, positive drug; TWBXD-L/M/H, low, medium, and high doses of Tianwang Buxin Dan. **(A)** Latency to first black stool (min). **(B)** Distal colonic transit time (min). **(C)** Carbon expulsion rate (%). Data are expressed as mean ± SD (*n* = 8). Statistical analysis was performed using one-way ANOVA followed by Tukey's multiple-comparison test or Dunnett's test, as appropriate. Detailed pairwise comparisons and significance symbols are provided in Supplementary Table S7, where *P < 0.05 compared with the control group and #*P* < 0.05 compared with the model group.

### 3.7 TWBXD improves anxiety- and depression-like behaviors in constipated rats

To evaluate the therapeutic effects of TWBXD on emotional disturbances associated with functional constipation, anxiety- and depression-like behaviors were assessed using the open field test (OFT) and the sucrose preference test (SPT).

In the SPT, the model group showed a marked decrease in sucrose preference compared to the control group (*p* < 0.05), indicating the presence of anhedonia and depressive-like behavior. Treatment with TWBXD at medium and high doses significantly reversed this effect, improving sucrose preference levels (*p* < 0.05 vs. model) (Figure 7A).

**Figure 7:**
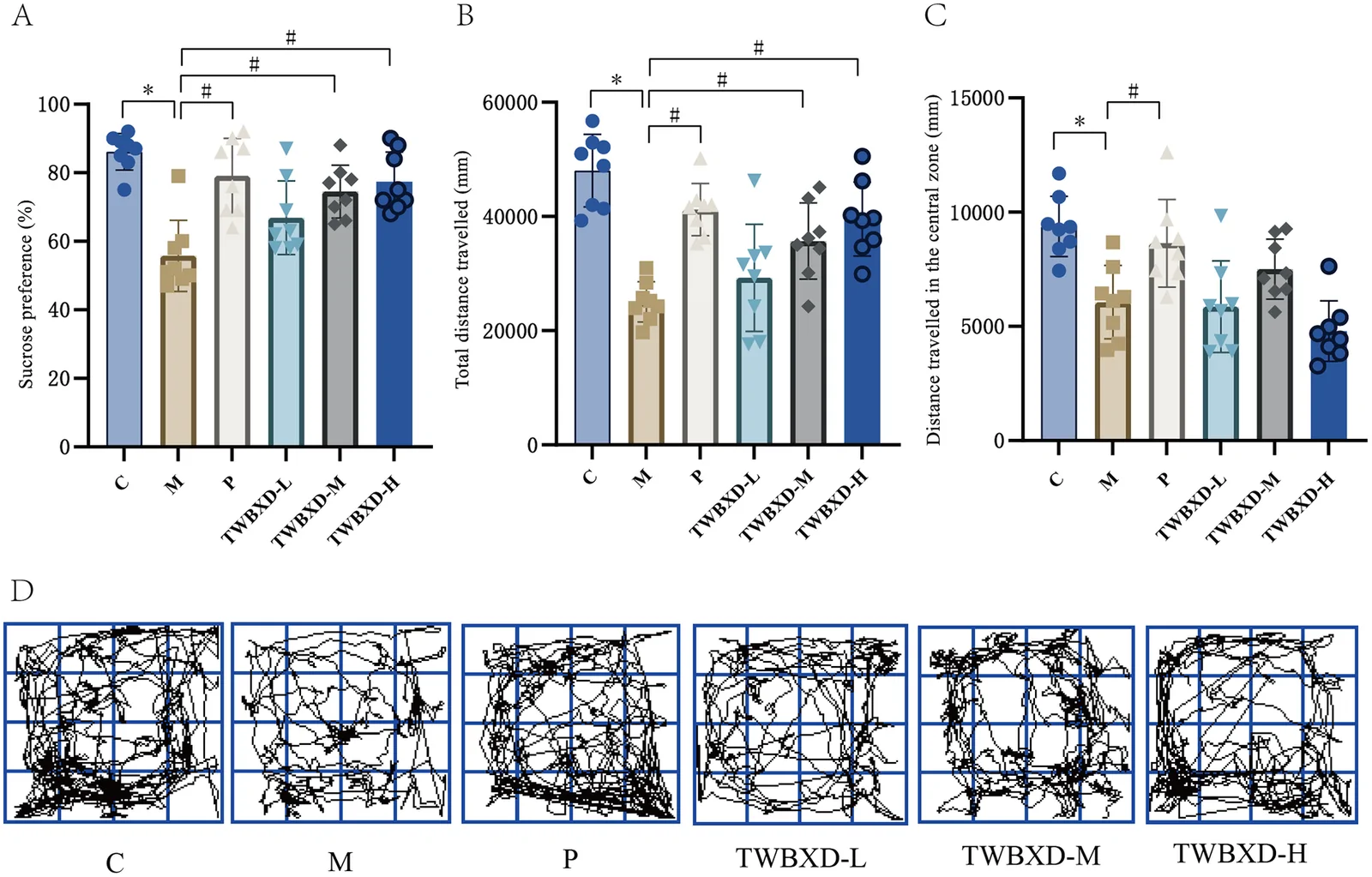
Effects of TWBXD on anxiety and depression like behaviors in rats. C, control group; M, model group; P, positive drug; TWBXD-L/M/H, low, medium, and high doses of Tianwang Buxin Dan. **(A)** Sucrose preference percentage. **(B)** Total distance travelled in the open-field test. **(C)** Distance travelled in the central zone of the open field. **(D)** Representative locomotor traces for each group; panel **(D)** is shown for visual illustration and was not subjected to separate statistical analysis. Quantitative statistical analyses of open-field performance are shown in panels **(B)** and **(C)** Data in panels **(A–C)** are expressed as mean ± SD (*n* = 8). Statistical analysis was performed using one-way ANOVA followed by Tukey's multiple-comparison test or Dunnett's test, as appropriate. *P < 0.05 compared with the control group; #*P* < 0.05 compared with the model group.

In the OFT, the model group exhibited significantly reduced total distance travelled and distance travelled in the central zone compared with the control group (*p* < 0.05), reflecting elevated anxiety-like behavior. TWBXD treatment, especially at high dose, significantly increased locomotor activity and central-zone exploration compared with the model group (*p* < 0.05), suggesting an anxiolytic-like effect (Figures 7B,7C). Representative locomotor traces for each group are shown in Figure 7D.

Overall, both behavioral assays demonstrated that TWBXD alleviates anxiety- and depression-like behaviors in rats with functional constipation, with the high-dose group exhibiting the greatest therapeutic efficacy.

### 3.8 TWBXD ameliorates pathological damage in colonic and hippocampal tissues of constipated rats with emotional disorders

Colonic histology (H&E staining): In the control group, colonic mucosa displayed abundant glands with intact U-shaped crypts and well-defined goblet cells; epithelial cells were tightly arranged with no signs of inflammatory infiltration. In contrast, the model group showed severe mucosal disruption, glandular loss, disappearance of crypts and goblet cells, diffuse inflammatory cell infiltration, structural disorganization, and submucosal edema. TWBXD treatment, especially at higher doses, markedly alleviated these pathological alterations, as evidenced by reduced mucosal edema, restored glandular alignment, and diminished inflammatory infiltration. The high-dose TWBXD group exhibited the most pronounced histological improvement, and the key pathological regions are indicated by arrowheads in Figure 8A.

**Figure 8:**
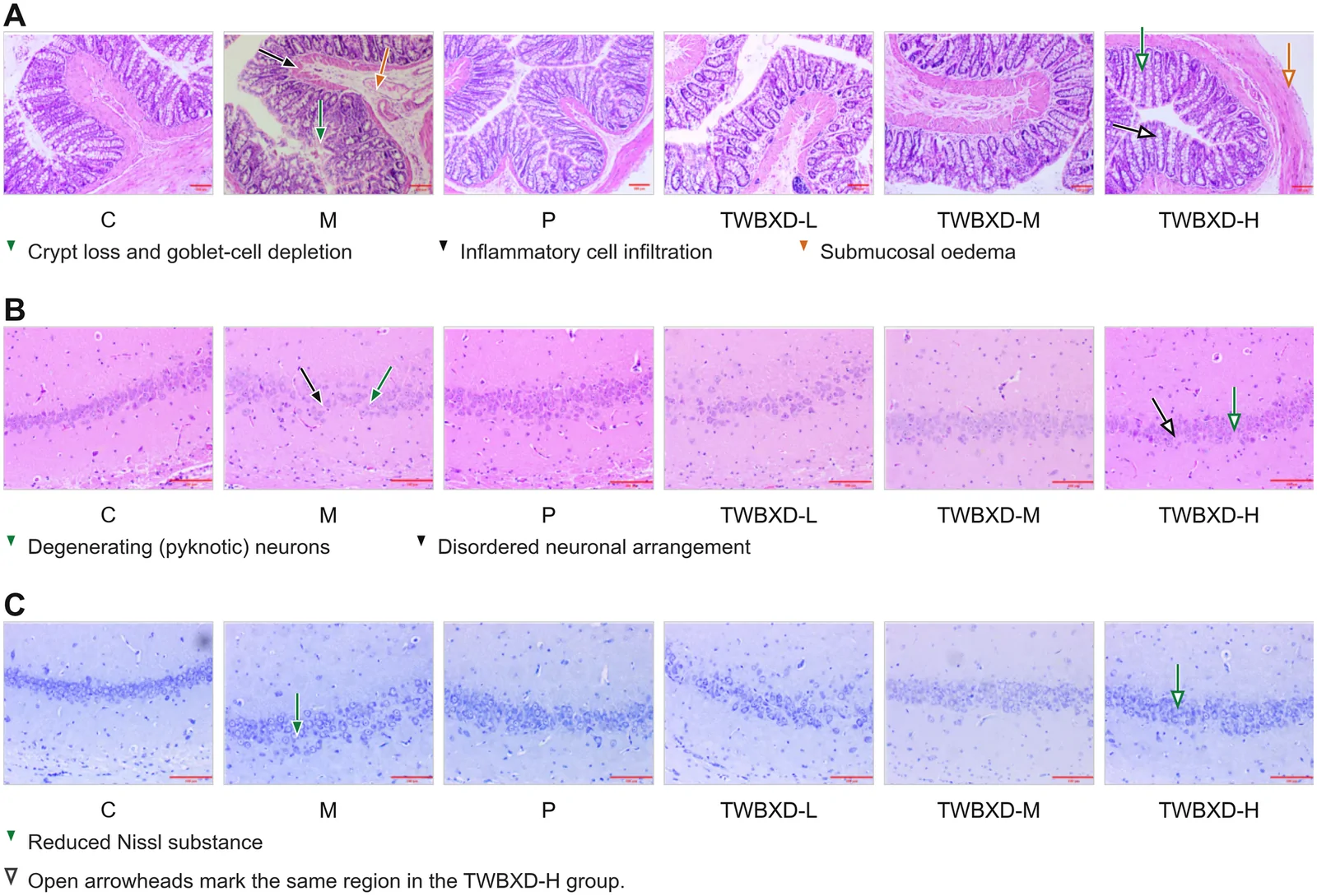
Histological changes in colonic and hippocampal tissues in each group (x100). C, control group; M, model group; P, positive drug; TWBXD-L/M/H, low, medium, and high doses of Tianwang Buxin Dan. **(A)** Hematoxylin-eosin (H&E) staining of distal colon. Arrowheads indicate the main pathological regions: green arrowheads, crypt loss and goblet-cell depletion; black arrowheads, inflammatory cell infiltration; orange arrowheads, submucosal oedema. **(B)** H&E staining of the hippocampal CA1 region. Green arrowheads indicate degenerating (pyknotic) neurons, and black arrowheads indicate disordered neuronal arrangement. **(C)** Nissl staining of the hippocampal CA1 region. Green arrowheads indicate reduced Nissl substance. Open arrowheads indicate the corresponding regions in the TWBXD-H group, showing alleviation of the pathological changes.

Hippocampal histology (H&E staining): In the control group, hippocampal neurons appeared round and well-organized, with intact structures and evenly stained nuclei, without signs of edema or inflammation. The model group exhibited severe neurodegenerative changes, including disordered neuronal arrangement, shrunken and dark-staining nuclei, and evidence of necrosis. TWBXD, particularly at high doses, partially restored hippocampal architecture with more regularly arranged neurons, clearer nucleoli, and reduced edema and inflammatory infiltration compared to the model group, and the representative regions showing neuronal degeneration and disordered arrangement are indicated by arrowheads in Figure 8B.

Nissl staining of the hippocampus: In the control group, CA1 neurons showed abundant, well-organized blue granular Nissl bodies. The model group displayed a marked reduction and disorganization of Nissl substance, indicating neuronal injury. TWBXD, especially at high doses, significantly increased the number and structural integrity of Nissl bodies compared to the model group, suggesting improved neuronal protein synthesis capacity and reduced neurodegeneration. The regions showing reduced Nissl substance are indicated by arrowheads in Figure 8C.

### 3.9 TWBXD improves mitochondrial ultrastructure in colonic and hippocampal tissues of constipated rats

Colonic tissue ultrastructure: In the control group, colonic epithelial mitochondria displayed regular morphology, moderate size, and diverse shapes (round, oval, rod-like), with well-organized alignment. The inner and outer membranes were smooth and intact, and cristae were clearly folded, densely arranged, and morphologically uniform. Mitochondrial matrices exhibited homogeneous electron density without signs of vacuolization or cristae rupture. In contrast, the model group showed marked mitochondrial injury, including swelling, irregular morphology, membrane disruption, disorganized or absent cristae, and abundant vacuole formation with myelin-like structures, indicating impaired oxidative phosphorylation. TWBXD treatment dose-dependently mitigated these ultrastructural injuries. The high-dose TWBXD group, similar to the positive control, showed restored cristae organization, reduced membrane rupture, and normalized electron density in the matrix, approximating control group levels (Figure 9A).

**Figure 9:**
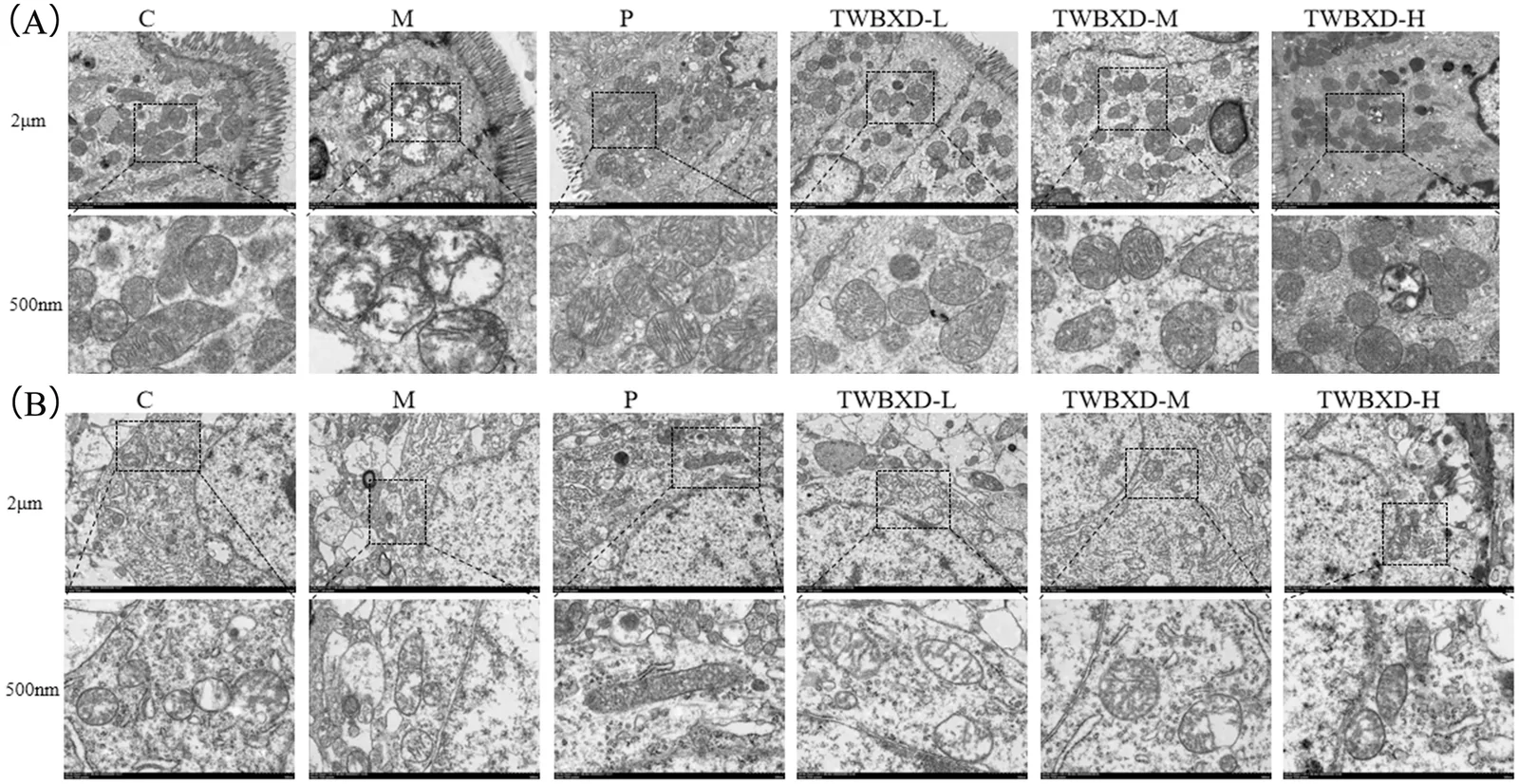
Ultrastructural changes in colon epithelium and hippocampal neurons observed by transmission electron microscopy. C, control group; M, model group; P, positive drug; TWBXD-L/M/H, low, medium, and high doses of Tianwang Buxin Dan. **(A)** Colon tissue; **(B)** Hippocampal tissue; Upper panels, ×10 000; scale ba*r* = 2 µm. Lower panels, enlarged views of the boxed regions, ×40 000; scale ba*r* = 500 nm.

Hippocampal tissue ultrastructure: In control rats, hippocampal neurons exhibited round or oval nuclei with intact nuclear membranes and uniform perinuclear spacing. Mitochondria had continuous inner and outer membranes, with clearly defined and well-organized cristae and homogeneous electron-dense matrices. Rough endoplasmic reticulum (RER) appeared well-aligned with intact ribosomes. In the model group, neuronal nuclei became irregular, mitochondrial membranes showed discontinuity and rupture, cristae were fragmented or diminished, and mitochondria were swollen with extensive vacuolization. Some mitochondria were enclosed by vacuoles forming autophagic vesicles, suggesting dysregulated autophagy. RER structures were disrupted, with dilated, fractured, or twisted profiles, and ribosomal detachment. TWBXD intervention, particularly at high doses, preserved nuclear integrity, reduced mitochondrial swelling and vacuolization, increased cristae number and organization, and normalized RER morphology. The high-dose TWBXD group showed ultrastructural improvements similar to those of the positive control group (Figure 9B).

### 3.10 TWBXD modulates the PI3K/Akt/mTOR signaling pathway in colonic and hippocampal tissues

To elucidate the molecular mechanisms underlying TWBXD’s therapeutic effects, we examined the expression of key proteins in the PI3K/Akt/mTOR signaling pathway in colonic and hippocampal tissues using Western blot analysis.

Colonic tissue: In the model group, the expression levels of PI3Kp85, phosphorylated Akt (p-Akt), and phosphorylated mTOR (p-mTOR) were significantly decreased compared with the control group (*p* < 0.01; Figure 10A), indicating pathway inhibition. TWBXD treatment significantly increased the expression of PI3Kp85, p-Akt, and p-mTOR in a dose-dependent manner (*p* < 0.01), with the high-dose group showing the most prominent activation. The positive drug group also showed increased expression of these proteins, with values between those of the low- and high-dose TWBXD groups (Figure 10A). This may be related to the known regulatory effects of fluoxetine on this signaling pathway, whereas mosapride lacks established involvement.

**Figure 10:**
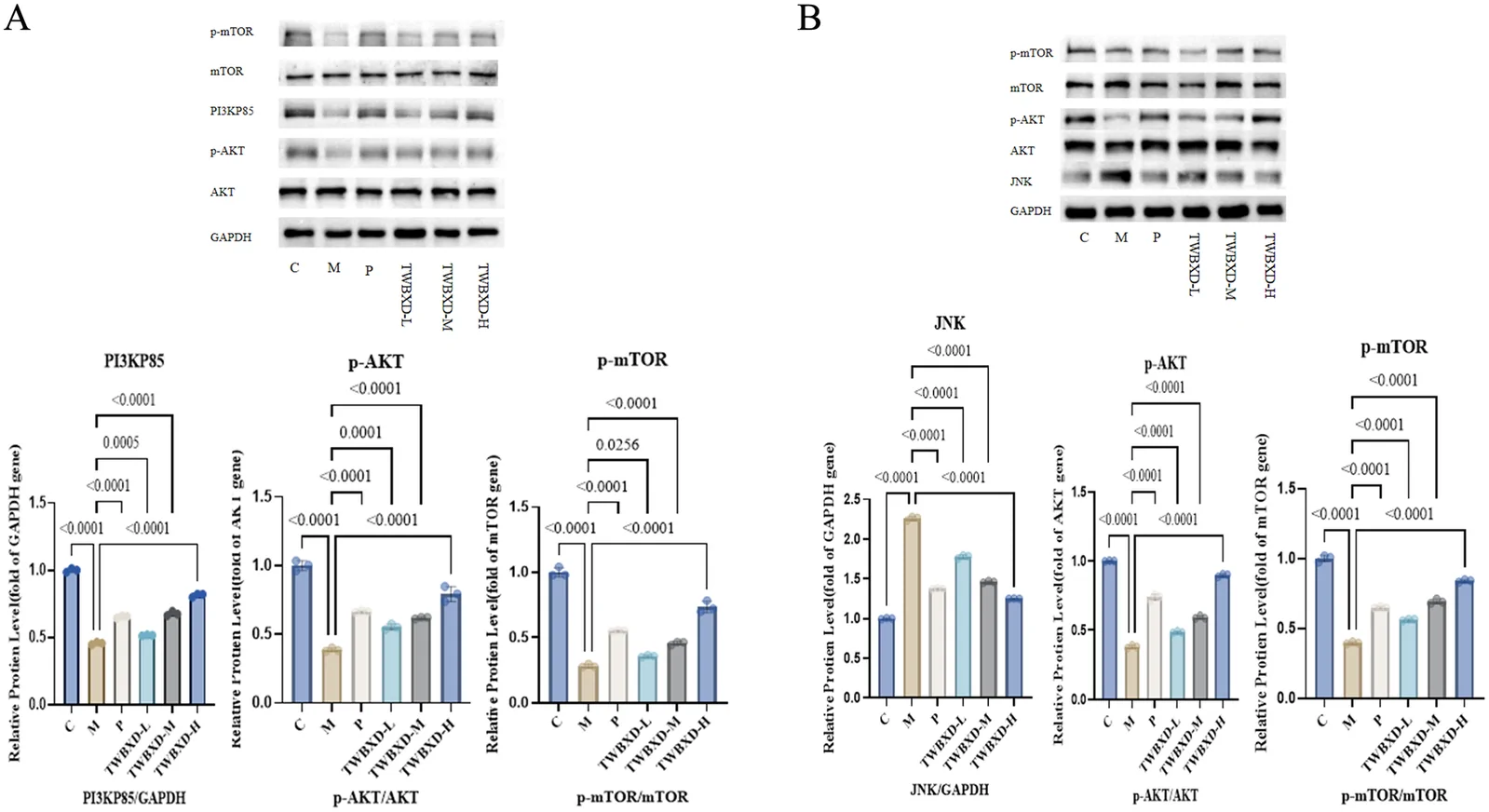
TWBXD modulates PI3K/Akt/mTOR-related signaling in colon and hippocampus tissue. C, control group; M, model group; P, positive drug; TWBXD-L/M/H, low, medium, and high doses of Tianwang Buxin Dan. p-Akt: phosphorylated Akt; p-mTOR: phosphorylated mTOR. **(A)** Representative Western blot bands and densitometric quantification of PI3K p85, p-Akt/AKT, and p-mTOR/mTOR in colonic tissue. **(B)** Representative Western blot bands and densitometric quantification of JNK/GAPDH, p-Akt/AKT, and p-mTOR/mTOR in hippocampal tissue. Quantitative data are expressed as mean ± SD from three independent replicates (*n* = 3 per group). Representative blots are shown above the corresponding quantification graphs. Statistical comparisons were performed using one-way ANOVA followed by *post hoc* multiple-comparison tests. Exact *p*-values are shown above the bars, and *p* < 0.05 was considered statistically significant.

Hippocampal tissue: In the model group, JNK expression was significantly elevated, while p-Akt and p-mTOR levels were markedly reduced (*p* < 0.01; Figure 10B), suggesting activation of pro-apoptotic signaling and impairment of neuroprotective mechanisms. TWBXD administration suppressed JNK expression and enhanced the phosphorylation of Akt and mTOR in a dose-dependent manner (*p* < 0.01). Similar trends were observed in the positive drug group, suggesting the involvement of PI3K/Akt/mTOR signaling in TWBXD-associated neuroprotective effects.

### 3.11 TWBXD regulates PI3K/Akt/mTOR- and JNK-related mRNA expression in colonic and hippocampal tissues

Quantitative real-time PCR (qPCR) was performed to assess the transcriptional regulation of PI3K/Akt/mTOR-related genes in colonic tissue and Akt, mTOR, and JNK-related gene expression in hippocampal tissue. The results were consistent with the protein expression trends observed by Western blot analysis.

In colonic tissue, the mRNA expression levels of PI3K, Akt, and mTOR were significantly increased following TWBXD treatment (*p* < 0.05), showing a dose-dependent enhancement (Figure 11A).

**Figure 11:**
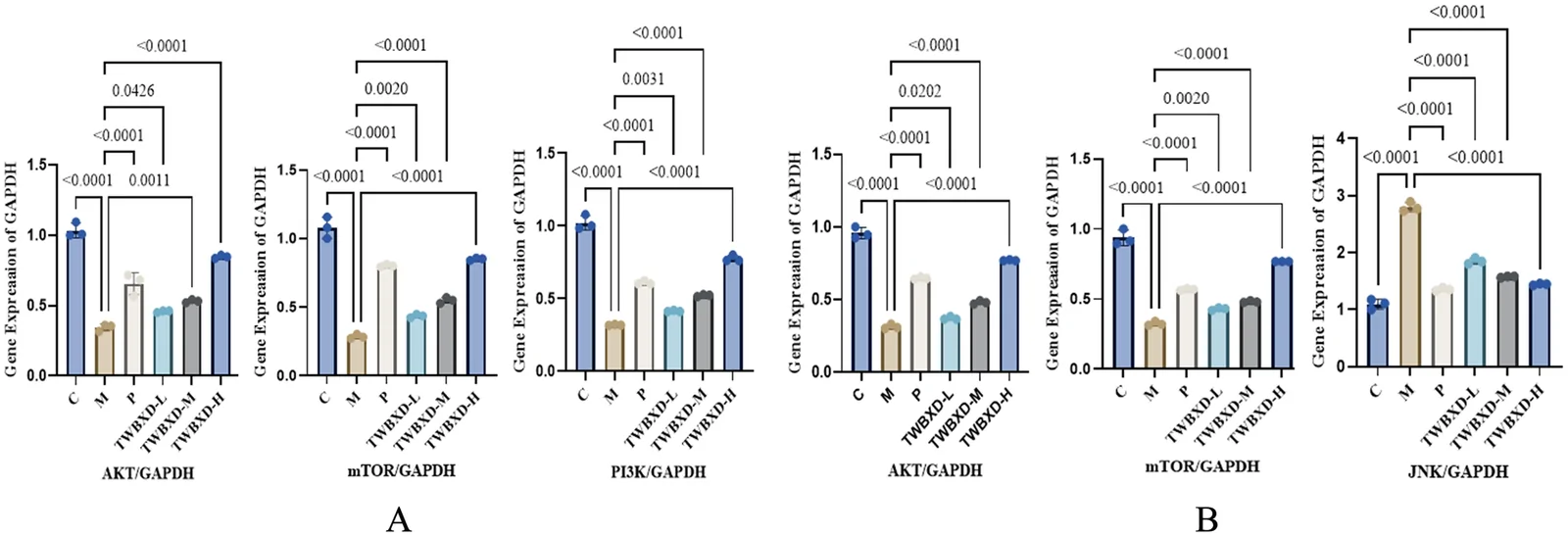
TWBXD regulates PI3K/Akt/mTOR- and JNK-related gene expression in colon and hippocampus. C, control group; M, model group; P, positive drug; TWBXD-L/M/H, low, medium, and high doses of Tianwang Buxin Dan. **(A)** Relative mRNA levels of Akt, mTOR, and PI3K in distal-colon tissue, quantified by qPCR and normalised to GAPDH. **(B)** Relative mRNA levels of Akt, mTOR, and JNK in hippocampal tissue, normalised to GAPDH. Statistical comparisons (one-way ANOVA) are indicated above the bars; exact *p*-values (< 0.05) are displayed for significant differences. A two-tailed *p* < 0.05 was considered statistically significant.

In hippocampal tissue, TWBXD administration upregulated the mRNA levels of Akt and mTOR (*p* < 0.05), while JNK mRNA expression was markedly reduced compared to the model group. The reduction in JNK expression exhibited a negative correlation with TWBXD dosage, indicating a dose-dependent regulatory effect (Figure 11B).

### 3.12 TWBXD shows good safety profile in rats with functional constipation and emotional disturbances

To assess the safety of TWBXD *in vivo*, serum biochemical parameters were measured following high-dose administration. No significant differences were observed in liver, kidney, or cardiac function indicators between the high-dose group and the control group (*p* > 0.05; Table 4), indicating that TWBXD did not induce overt organ toxicity at the tested dosage.

**Table 4:** Comparison of serum biochemical indicators between control and TWBXD-H groups.

Indicator	Control	TWBXD-H
ALT (U/L)	35.82 ± 3.28	32.99 ± 4.27
AST (U/L)	96.16 ± 7.11	96.95 ± 8.73
UREA (mmol/L)	5.81 ± 1.06	5.81 ± 1.08
CREA (*μ*mol/L)	46.61 ± 3.07	47.76 ± 4.23
CK (U/L)	441.60 ± 76.21	442.61 ± 74.99

These findings suggest that TWBXD is well tolerated in the functional constipation rat model with comorbid emotional disturbances.

## 4 Discussion

In this study, we employed an integrated approach combining network pharmacology, molecular docking, and animal experiments to investigate the pharmacological mechanisms of TWBXD in treating FC with emotional disturbances. Recent advances in single-cell omics have provided new opportunities to dissect the mechanisms of traditional Chinese medicines at cellular resolution and to clarify how complex herbal formulas regulate multicellular biological networks (33). TWBXD significantly improved defecation parameters and mitigated anxiety- and depression-like behaviors in rats. These effects were associated with preserved mucosal and neuronal structures and upregulation of PI3K/Akt/mTOR signaling in colonic and hippocampal tissues. The parallel involvement of peripheral and central tissues raises the possibility that gut–brain communication contributes to the therapeutic effects of TWBXD, although a direct gut–brain link was not established in the present study. The present findings suggest that TWBXD can improve intestinal motility, protect colonic and hippocampal tissue architecture, and modulate the activation of the PI3K/Akt/mTOR signaling pathway.

The PI3K/Akt/mTOR pathway is a key intracellular signaling cascade that regulates cell survival, proliferation, and metabolism, and is critically involved in maintaining both gastrointestinal and neural homeostasis (34). In the gut, its downregulation has been implicated in impaired motility and epithelial barrier dysfunction, partly through promoting apoptosis of interstitial cells of Cajal (ICCs), as observed in slow-transit constipation (STC) models (35, 36). Conversely, pathway activation supports ICC survival and promotes colonic transit. In the central nervous system, this pathway is abundantly expressed in the hippocampus and prefrontal cortex, where it enhances neuroplasticity, suppresses autophagy, and exerts antidepressant effects (37, 38). Agents like ketamine rapidly alleviate depressive symptoms via mTOR-mediated synaptic remodeling (39). Together, these findings indicate that PI3K/Akt/mTOR is a central molecular node involved in both gastrointestinal and central nervous system processes, and may represent a shared signaling pathway relevant to gastrointestinal and psychiatric manifestations. In our network pharmacology analysis, 308 overlapping targets of TWBXD were identified in the treatment of constipation with emotional disturbances. Core targets included Akt1, IL-6, TNF, and TP53, among others. Although the PI3K-Akt pathway was not the highest-ranked KEGG term, it was significantly enriched and was selected for mechanistic validation because it converged with several lines of evidence, including the presence of AKT1 among the PPI hub targets, the established role of PI3K/Akt/mTOR signaling in intestinal motility and epithelial barrier repair, and its involvement in hippocampal neuroplasticity and mood regulation. These results suggest that TWBXD may engage the PI3K/Akt/mTOR axis as one of several relevant signaling pathways rather than as the sole or top-ranked pathway.

### 4.1 Therapeutic effects of TWBXD on gastrointestinal dysfunction and emotional disturbances

Following four weeks of treatment with TWBXD, rats exhibited significant improvements in both gastrointestinal and behavioral parameters. TWBXD enhanced gastrointestinal motility in a dose-dependent manner, as evidenced by accelerated charcoal meal transit, increased fecal moisture content, and reduced time to the first black stool. These effects were comparable to those of mosapride, with the high-dose group showing the most pronounced benefit. The improvement in colonic propulsion is consistent with previous findings that activation of mucosal 5-HT₄ receptors facilitates enteric neurotransmission and peristalsis (40). Hesperidin and quercetin, both major constituents of TWBXD, have been reported to enhance gastrointestinal motility by upregulating 5-HT₄ receptor expression and modulating the cAMP-PKA-Ca^2^⁺ pathway, thereby restoring interstitial cell of Cajal (ICC) function and accelerating transit in models of slow-transit constipation (41, 42).

In behavioral assessments, TWBXD-treated rats performed better in both the open-field and sucrose preference tests, indicating reductions in anxiety and depression like behaviors. Notably, the high-dose group produced effects comparable to fluoxetine, suggesting a considerable psychotropic potential. Luteolin, identified as a major bioactive compound in our network pharmacology analysis, has been shown to promote hippocampal neurogenesis and enhance synaptic plasticity by activating the BDNF/TrkB-Akt signaling pathway (43, 44). This cascade is functionally linked to mTOR activation, a mechanism underlying the rapid antidepressant effects of agents such as ketamine (45, 46). In addition, quercetin has demonstrated anxiolytic and antidepressant effects via modulation of neuroinflammatory pathways and hippocampal BDNF signaling, providing further support for TWBXD's neuroregulatory properties (47). Such enhancement of neuroplasticity likely provides the structural foundation underlying the anxiolytic and antidepressant effects recorded in our behavioral assays.

Collectively, these findings underscore the dual therapeutic action of TWBXD in promoting intestinal motility and relieving emotional symptoms.

### 4.2 Restoration of colonic and hippocampal architecture

TWBXD administration restored tissue architecture in both the intestine and the brain, indicating that peripheral barrier integrity and central neuroprotection were both preserved. Histological analysis of the colon revealed restoration of crypt structure, increased goblet-cell density, and reduced inflammatory infiltration. These changes coincided with the upregulation of tight-junction protein claudin-1 and mucin-related gene expression, indicating reinforcement of epithelial barrier function. PI3K/Akt signaling plays a central role in the transcriptional regulation of claudins and mucins under physiological and injury-repair conditions (48, 49). Compounds abundant in TWBXD, such as quercetin and luteolin, have been shown to activate this pathway and enhance epithelial integrity in models of intestinal inflammation and stress-related dysfunction (50, 51).

Transmission electron microscopy further revealed that TWBXD preserved mitochondrial ultrastructure, including intact cristae and reduced autophagic vacuole formation, in both colonic epithelial cells and hippocampal neurons. Mitochondrial dysfunction is a key pathological feature in chronic inflammatory and stress-related disorders, often leading to epithelial barrier collapse and synaptic degeneration due to impaired ATP production and oxidative stress (52). The observed restoration of mitochondrial structure is supported by biochemical evidence showing TWBXD-associated phosphorylation of Akt and mTOR in colonic and hippocampal tissues and concurrent inhibition of JNK expression in the hippocampus. This signaling pattern promotes mitochondrial biogenesis, suppresses excessive autophagy, and reduces stress-related apoptotic pathways, which together help maintain cellular energy homeostasis (53, 54).

In the hippocampus, TWBXD alleviated neuronal abnormalities such as nuclear condensation and cytoplasmic edema, while Nissl staining demonstrated increased ribosomal density and preserved neuronal cytoarchitecture. These neuroprotective effects likely reflect the combined influence of mitochondrial preservation and neurotrophic signaling. Luteolin and quercetin have been reported to enhance BDNF/TrkB-Akt signaling in the hippocampus, promoting neuronal survival, synaptic plasticity, and dendritic remodeling under conditions of chronic stress and inflammation (55, 56). Given the role of BDNF-mTOR signaling in emotional regulation, these structural improvements may underpin the behavioral normalization observed in TWBXD-treated animals.

Taken together, these findings indicate that TWBXD is associated with the preservation of epithelial and neuronal structure, accompanied by activation of the PI3K/Akt/mTOR pathway, inhibition of stress-associated JNK signaling, and maintenance of mitochondrial morphology. These coordinated changes may contribute to strengthening gut barrier integrity and stabilizing hippocampal neurons, thereby providing a structural basis for the therapeutic effects observed against both gastrointestinal dysfunction and emotional disturbances.

### 4.3 Parallel regulation of PI3K/Akt/mTOR and JNK signaling in colonic and hippocampal tissues: a potential gut–brain link

The present study showed that TWBXD was associated with activation of PI3K/Akt/mTOR signaling in colonic and hippocampal tissues and with suppression of JNK signaling in the hippocampus. These changes occurred in parallel in the periphery and the central nervous system. Although our dataset cannot establish that the two compartments are functionally connected, this pattern may reflect a coordinated shift from stress-associated injury toward tissue repair, neuronal protection, and functional recovery, and is consistent with the concept of gut–brain axis regulation. In the intestine, PI3K/Akt signaling is involved in preservation of epithelial barrier integrity and mucosal repair. Previous studies have shown that TFF3/TLR2-dependent activation of PI3K/Akt enhances the expression of tight junction-associated proteins, including ZO-1, occludin, and claudin-1, thereby improving epithelial barrier function and reducing permeability-related injury (57). In addition, reduced Akt-mediated survival signaling in enteric neurons has been linked to myenteric neuronal apoptosis, loss of inhibitory neurons, and gastrointestinal motility abnormalities, whereas restoration of this pathway rescues enteric neuropathy and improves motility-related phenotypes (58). Akt-dependent phosphorylation of enteric neuronal NO synthase (nNOS) also regulates nitrergic neurotransmission and smooth-muscle relaxation in the gut, further indicating that this pathway participates in ENS–smooth muscle coordination (59). Therefore, in the present model, activation of PI3K/Akt/mTOR signaling in the colon may not only reflect attenuation of local injury, but also suggest recovery of mucosal barrier homeostasis, enteric neuronal support, and intestinal motility regulation.

The hippocampal findings can be interpreted within the same mechanistic framework. PI3K/Akt is a classical pro-survival pathway in neurons, and Akt activation has been shown to protect primary hippocampal neurons against hypoxia- or nitric oxide-induced apoptosis (60). In depression-related models, suppression of hippocampal PI3K/Akt/mTOR signaling is associated with emotional abnormalities, whereas restoration of this pathway contributes to antidepressant-like behavioral effects (61). Moreover, chronic stress impairs hippocampal mTORC1 signaling, leading to reduced synaptic protein synthesis, synapse loss, and depression-like behaviors, while pharmacological restoration of this pathway is associated with recovery of synaptic proteins such as PSD-95 and synapsin I (62, 63). Thus, the restoration of PI3K/Akt/mTOR signaling in the hippocampus observed in the present study may help explain the improved hippocampal ultrastructure and the amelioration of anxiety- and depression-like behaviors, because this pathway is closely linked to neuronal survival, synaptic plasticity, and emotional modulation.

Importantly, inhibition of JNK signaling may complement PI3K/Akt/mTOR activation in the hippocampus. JNK is a stress-activated kinase that is rapidly engaged by inflammatory and psychological stressors, and hippocampal JNK activation has been shown to impair stress-related learning and memory (64). More recent work further demonstrated that constitutive JNK activation is sufficient to induce depression-like behaviors and is accompanied by abnormal dendritic spine development and PSD95 downregulation (65). Conversely, selective inhibition of JNK in adult-born hippocampal granule cells promotes neurogenesis and alleviates anxiety- and depressive-like behaviors (66). Taken together, our findings suggest that TWBXD exerts an integrated pharmacological action in which PI3K/Akt/mTOR-dependent restorative signaling and JNK-associated stress signaling are coordinately rebalanced. Such a dual regulation pattern may simultaneously favor colonic barrier repair and motility recovery while protecting hippocampal neuronal integrity and synaptic plasticity. Whether these peripheral and central actions are functionally coupled through the gut–brain axis remains to be determined.

### 4.4 Limitations

This study has several limitations. Although TWBXD was consistently associated with PI3K/Akt/mTOR activation in colonic and hippocampal tissues and with suppression of JNK signaling in the hippocampus, we did not use pathway-specific inhibitors or genetic manipulation to establish causality. In particular, no PI3K inhibitor (LY294002), mTOR inhibitor (rapamycin), or JNK inhibitor (SP600125) was administered, either alone or in combination with TWBXD. Therefore, the signaling changes reported here should be interpreted as mechanistic associations rather than definitive mediation, and we cannot exclude contributions from additional signaling networks. Future studies will apply pharmacological inhibition and genetic approaches to confirm pathway dependence and to clarify the potential contributions of other signaling networks such as AMPK and MAPK.

In addition, although signaling changes were examined in both colonic and hippocampal tissues, no direct mediator of gut–brain communication was assessed in this study. Microbiota composition, microbial metabolites, intestinal permeability, vagal activity, and circulating neuroendocrine or inflammatory mediators were not measured, and no vagotomy, microbiota depletion, or fecal microbiota transplantation experiment was performed. The present data therefore demonstrate parallel peripheral and central responses to TWBXD rather than a functional connection between the gut and the brain, and the gut–brain axis interpretation should be regarded as hypothesis-generating rather than as a demonstrated mechanism.

In addition, our conclusions were derived from a single rat model of constipation comorbid with emotional disturbance, and the generalizability to other etiological subtypes of functional constipation and broader affective phenotypes remains to be determined. Further validation in independent models and larger cohorts will help strengthen the robustness and translational relevance of our findings.

Finally, downstream readouts related to autophagy and oxidative stress were not directly quantified in the current study and warrant further investigation in future work.

## 5 Conclusion

In conclusion, TWBXD significantly ameliorated constipation-related gastrointestinal dysfunction and anxiety- and depression-related behaviors in rats, while protecting colonic and hippocampal structure. These effects were associated with activation of PI3K/Akt/mTOR signaling in colonic and hippocampal tissues and with modulation of JNK signaling in the hippocampus. Whether these peripheral and central changes are functionally coupled through the gut–brain axis, and whether PI3K/Akt/mTOR activation mediates the therapeutic effects, remain to be established. The present findings provide experimental evidence for the multi-target pharmacological actions of TWBXD and support its further investigation as an integrative therapy for functional constipation with emotional comorbidities.

## Data Availability

The raw data supporting the conclusions of this article will be made available by the authors, without undue reservation.

## Glossary

Akt: protein kinase B
ALT: alanine aminotransferase
ANOVA: analysis of variance
AST: aspartate aminotransferase
BATMAN-TCM: Bioinformatics Analysis Tool for Molecular Mechanism of Traditional Chinese Medicine
BCA: bicinchoninic acid
BDNF: brain-derived neurotrophic factor
BP: biological process
CA1: Cornu Ammonis 1
CC: cellular component
CE: collision energy
CK: creatine kinase
CREA: creatinine
CTD: Comparative Toxicogenomics Database
CUMS: chronic unpredictable mild stress
DL: drug-likeness
DP: declustering potential
ESI: electrospray ionization
FC: functional constipation
GAPDH: glyceraldehyde-3-phosphate dehydrogenase
GO: Gene Ontology
H&E: hematoxylin and eosin
ICC: interstitial cells of Cajal
IL-6: interleukin-6
IL-17: interleukin-17
JNK: c-Jun N-terminal kinase
KEGG: Kyoto Encyclopedia of Genes and Genomes
MAPK: mitogen-activated protein kinase
MF: molecular function
MRM: multiple reaction monitoring
MS/MS: tandem mass spectrometry
mRNA: messenger RNA
mTOR: mechanistic target of rapamycin
OB: oral bioavailability
OFT: open-field test
OMIM: Online Mendelian Inheritance in Man
PBS: phosphate-buffered saline
PDB: Protein Data Bank
PI3K: phosphoinositide 3-kinase
PPI: protein-protein interaction
qPCR: quantitative real-time polymerase chain reaction
RER: rough endoplasmic reticulum
SD: standard deviation
SPF: specific-pathogen-free
SPT: sucrose preference test
STRING: Search Tool for the Retrieval of Interacting Genes/Proteins
TCMSP: Traditional Chinese Medicine Systems Pharmacology Database and Analysis Platform
TEM: transmission electron microscopy
TNF: tumor necrosis factor
TWBXD: Tianwang Buxin Dan
UHPLC: ultra-high-performance liquid chromatography
UHPLC-MS/MS: ultra-high-performance liquid chromatography-tandem mass spectrometry
UniProt: Universal Protein Resource
WB: western blot
