# Supplementary material for: Tianwang Buxin Dan regulated PI3K/Akt/mTOR signaling pathway to improve constipation and emotional disorders in rats
Source: Front Med (Lausanne). 2026 Oct 14;13:1942569. doi: 10.3389/fmed.2026.1942569 (PMC13717440; doi:10.3389/fmed.2026.1942569)
Supplement: Supplementary file 1 [file Table1.docx]

Supplementary Material

**Supplementary tables**

**Table S1. Experimental grouping and drug administration of rats in each group.**

| **Group (n = 8/group)** | **Modeling** | **Drug Administration** |
| --- | --- | --- |
| **Control** | No treatment was given; animals had free access to food and water. The same administration route was applied as in the medication groups, with an equal volume of physiological saline. | |
| **Model** | Rats were housed in isolation and subjected to chronic unpredictable mild stress (CUMS) to establish a stress model for 6 weeks. Beginning in the third week of modeling, compound diphenoxylate was administered by gavage at a dose of 10 mg/kg for 4 consecutive weeks, during which the CUMS procedure was maintained. The total intervention period lasted 6 weeks. | Gavage with physiological saline |
| **Positive** |  | Mosapride 1.351 mg/kg/d + Fluoxetine 1.58 mg/kg/d, gavage for 4 weeks |
| **TWBXD-L** |  | TWBXD 7.88 g/kg/d, gavage for 4 weeks |
| **TWBXD-M** |  | TWBXD 15.75 g/kg/d, gavage for 4 weeks |
| **TWBXD-H** |  | TWBXD 31.5 g/kg/d, gavage for 4 weeks |

**Table S2 Primer sequences used for quantitative real-time PCR. Mapk8 encodes JNK1.**

| **Gene** | **Forward primer (5′ → 3′)** | **Reverse primer (5′ → 3′)** |
| --- | --- | --- |
| Pik3r1 | GGCGAAGTCAAACATTGCGTCATC | GTGACATTGAGGGAGTCATTGTGC |
| Akt1 | TCATTGAGCGCACCTTCCAT | CTCCTGCCGTTTGAGTCCAT |
| Mtor | TGCCAACTACCTTCGGAACC | GCTCGCTTCACTTCAAACTCC |
| Mapk8 | GCCCGCGTCTCTGTTACTC | TCTCCCATGATGCACCCAAC |
| GAPDH | TCTCTGCTCCTCCCTGTTCT | ATCCGTTCACACCGACCTTC |

**Table S3 Normal reference ranges for routine serum biochemistry in adult rats**

| **Indicator set** | **Parameter** | **Unit** | **Normal range** |
| --- | --- | --- | --- |
| **Liver function** | ALT | U L⁻¹ | 21.53 – 61.75 |
|  | AST | U L⁻¹ | 41.47 – 195.65 |
| **Renal function** | UREA | mmol L⁻¹ | 3.50 – 8.11 |
|  | CREA (CR) | μmol L⁻¹ | 10.90 – 118.07 |
| **Cardiac enzymes** | CK | U L⁻¹ | 58.24 – 530.38 |

**Table S4. Top 10 hub genes of TWBXD in the treatment of FC with emotional disorders**

| Target Gene | Betweenness Centrality | Closeness Centrality | Degree |
| --- | --- | --- | --- |
| INS | 0.059999917 | 0.771689498 | 122 |
| AKT1 | 0.03603191 | 0.754464286 | 119 |
| IL6 | 0.025826453 | 0.731601732 | 111 |
| TNF | 0.022131004 | 0.725321888 | 110 |
| IL1B | 0.038594178 | 0.710084034 | 104 |
| TP53 | 0.021772401 | 0.698347107 | 104 |
| EGFR | 0.025269302 | 0.698347107 | 103 |
| ESR1 | 0.020806551 | 0.68699187 | 99 |
| STAT3 | 0.008422905 | 0.665354331 | 95 |
| JUN | 0.00878053 | 0.66798419 | 95 |

**Notes:** JUN was included among the top 10 hub genes and was selected for molecular docking because it is a canonical downstream effector of JNK/AP-1 signaling.

**Table S5. Top 10 bioactive components of TWBXD based on network topology**

| Active Compound | Betweenness Centrality | Closeness Centrality | Degree |
| --- | --- | --- | --- |
| quercetin | 0.386572249 | 0.366485014 | 73 |
| luteolin | 0.202881247 | 0.327250608 | 55 |
| beta-sitosterol | 0.072819553 | 0.305681818 | 39 |
| Salidroside | 0.11395553 | 0.28987069 | 27 |
| Stigmasterol | 0.124760098 | 0.293668122 | 25 |
| RUTIN | 0.126034853 | 0.306378132 | 22 |
| tanshinone iia | 0.054030204 | 0.299554566 | 15 |
| 3β-acetoxyatractylone | 0.030447669 | 0.279045643 | 12 |
| arachidonic acid | 0.041691112 | 0.26372549 | 11 |
| Gossypin | 0.055803239 | 0.29495614 | 11 |

**Table S6. Comparison of Fecal Water Content in Rats Before and After Treatment**

| Group | N | Pre-modeling | Post-modeling | Day 14 | Day 28 |
| --- | --- | --- | --- | --- | --- |
| Control | 8 | 46.67±6.50 | 45.50±6.72 | 46.17±3.54 | 43.83±1.47 |
| Model | 8 | 47.50±4.23 | 23.67±3.93^*^ | 25.50±3.01^*^ | 27.33±3.56^*^ |
| Positive | 8 | 46.33±5.88 | 22.50±4.76 | 41.83±1.84^#^ | 42.67±2.94^#^ |
| TWBXD-L | 8 | 48.67±5.20 | 23.17±3.25 | 27.67±4.37 | 32.83±4.17^#^ |
| TWBXD-M | 8 | 47.00±4.94 | 23.00±2.28 | 31.17±2.64^#^ | 40.17±3.13^#^ |
| TWBXD-H | 8 | 53.67±2.50 | 24.67±4.18 | 38.83±5.67^#^ | 42.17±2.48^#^ |

**Abbreviations:** C: control group; M: model group; P: positive drug; TWBXD-L/M/H: low, medium, and high doses of Tianwang Buxin Dan.

**Notes:** Data are presented as mean ± standard deviation (SD), *n* = 8 per group.
*^*^P* < 0.05 compared with the control group;
^#^*P* < 0.05 compared with the model group.

**Table S7. Comparison of Constipation-Related Indicators in Each Group After Treatment**

| Group | N | First black stool (min) | Distal colonic transit (min) | Carbon expulsion rate (%) |
| --- | --- | --- | --- | --- |
| Control | 8 | 290.33±29.06 | 204.50±13.77 | 91.33±3.89 |
| Model | 8 | 357.67±13.95^*^ | 273.17±13.93^*^ | 58.67±6.99^*^ |
| Positive | 8 | 300.33±12.69^#^ | 218.33±7.45 ^#^ | 87.65±4.44^#^ |
| TWBXD-L | 8 | 337.50±43.98 | 258.50±16.94 | 69.03±6.32^#^ |
| TWBXD-M | 8 | 317.50±44.42 | 225.33±10.87^#^ | 78.89±6.55^#^ |
| TWBXD-H | 8 | 304.83±60.68^#^ | 215.17±15.54^#^ | 85.21±2.11^#^ |

**Abbreviations:** C: control group; M: model group; P: positive drug; TWBXD-L/M/H: low, medium, and high doses of Tianwang Buxin Dan.

**Notes:** Data are presented as mean ± standard deviation (SD), *n* = 8 per group.
*^*^P* < 0.05 compared with the control group;
^#^*P* < 0.05 compared with the model group.

**Table S8. Targeted MRM quantification of eight selected marker constituents of TWBXD.**

| **Compound** | **MRM transition** | **Ion mode** | **Calibration range (ng/mL)** | **r** | **Content (ng/mL)** |
| --- | --- | --- | --- | --- | --- |
| Ferulic acid | 193.2 - 134.1 | [M−H]⁻ | 100–900 | 0.9966 | 310 |
| Polygalaxanthone III | 567.1 - 314.7 | [M−H]⁻ | 100–1000 | 0.9965 | 290 |
| Chlorogenic acid | 353.0 - 191.0 | [M−H]⁻ | 100–900 | 0.9920 | 264 |
| Rutin | 609.1 - 300.0 | [M−H]⁻ | 100–900 | 0.9959 | 217 |
| Naringenin | 271.0 - 119.1 | [M−H]⁻ | 100–900 | 0.9953 | 185 |
| Schisandrol A | 406.3 - 300.0 | [M+H]⁺ | 100–800 | 0.9945 | 125 |
| Luteolin | 285.0 - 133.0 | [M−H]⁻ | 200–900 | 0.9928 | 30.5 |
| Hyperin | 463.1 - 300.1 | [M−H]⁻ | 100–900 | 0.9899 | 3.23 |

**Notes:** Contents were calculated using external calibration curves constructed from MRM peak areas. r represents the weighted correlation coefficient of the calibration curve. The values are expressed as concentrations in the final sample solution. Values outside the calibration range are shown as estimated concentrations.

**Supplementary Figure S1. Complete STRING protein–protein interaction network of TWBXD-related common targets.**

**Notes:** The network was generated using STRING with Homo sapiens as the species and a minimum required interaction score >0.9. This complete network supports the core hub-target visualization shown in Figure 2C.
